# Wnt pathway inhibitors are upregulated in XLH dental pulp cells in response to odontogenic differentiation

**DOI:** 10.1038/s41368-022-00214-z

**Published:** 2023-02-27

**Authors:** Elizabeth Guirado, Cassandra Villani, Adrienn Petho, Yinghua Chen, Mark Maienschein-Cline, Zhengdeng Lei, Nina Los, Anne George

**Affiliations:** 1 Department of Oral Biology, University of Illinois Chicago, Chicago, IL USA; 2 Research Informatics Core, University of Illinois at Chicago, Chicago, IL USA; 3 Bioinformatics Scientist III, Ambry Genetics, Aliso, CA USA; 4 Genome Research Core, University of Illinois at Chicago, Chicago, IL USA

**Keywords:** Oral diseases, RNA sequencing

## Abstract

X-linked hypophosphatemia (XLH) represents the most common form of familial hypophosphatemia. Although significant advances have been made in the treatment of bone pathology, patients undergoing therapy continue to experience significantly decreased oral health-related quality of life. The following study addresses this persistent oral disease by further investigating the effect of DMP1 expression on the differentiation of XLH dental pulp cells. Dental pulp cells were isolated from the third molars of XLH and healthy controls and stable transduction of full-length human DMP1 were achieved. RNA sequencing was performed to evaluate the genetic changes following the induction of odontogenic differentiation. RNAseq data shows the upregulation of inhibitors of the canonical Wnt pathway in XLH cells, while constitutive expression of full-length DMP1 in XLH cells reversed this effect during odontogenic differentiation. These results imply that inhibition of the canonical Wnt pathway may contribute to the pathophysiology of XLH and suggest a new therapeutic strategy for the management of oral disease.

## Introduction

X-linked hypophosphatemia (XLH) represents the most common form of familial hypophosphatemia occurring in 1–5 per 100,000 annual births.^1–3^ Defective dentin, cementum, and alveolar bone contribute to the disease’s significant morbidity.^4–7^ Dental pulp necrosis in the absence of trauma or caries remains a significant long-term side-effect in individuals receiving therapy, with prevalence as high as 75% reported.^6,8–10^ These lesions present as spontaneous dental abscesses and can lead to more severe infections, tooth loss, occlusal disharmonies, and poor alveolar-dental development. The disorganized odontoblast cell layer and abnormal accumulation of non-collagenous extracellular matrix proteins in the XLH tooth suggest that defects in odontogenic differentiation may also be present in the disease.^7,11,12^

Odontoblast differentiation requires cell polarization and the formation of membrane domains and cell junctions that ensure the segregation and the unidirectional trafficking of molecules for mineralization.^13^ Canonical Wnt signaling is involved in tooth initiation and morphogenesis, correlating with odontoblast differentiation and dentin deposition.^14–16^ Despite a gradual decline in Wnt signaling with age, the conditional stabilization of beta-catenin in the adult pulp leads to dentin formation.^17,18^ The structural changes that accompany cytodifferentiation and tooth morphogenesis directly affect cell signaling and vice versa.^19^ E-cadherin is one component of adherens junctions necessary for palisade formation that is transcriptionally regulated by the Wnt pathway but also sequesters beta-catenin limiting its downstream Wnt pathway functions.^20^ The importance of the Wnt pathway in tooth development and regeneration has been well established; however, the status of Wnt signaling within the context of XLH remains unclear.^21^

Indication for deregulation of the Wnt pathway in XLH is implied from that seen in autosomal recessive hypophosphatemic rickets, a disorder phenotypically similar to XLH resulting from dentin matrix protein 1 (DMP1) loss-of-function.^22^ Expression of canonical Wnt pathway inhibitors, such as the secreted frizzled-related protein 4 (sFRP-4), have been reported in *Dmp1* knockout mice.^23^ sFRP-4 has been associated with Wnt Family Member 5A (*WNT5A*) expression and noncanonical Wnt signaling pathway activity, as well as, activation of bone morphogenic protein (BMP) signaling and sclerostin (*SOST*) gene expression, contributing to decreased bone formation.^24^ Indeed, patients with XLH are reported to have higher concentrations of circulating sclerostin.^25^

Our group previously reported impaired matrix mineralization in XLH dental pulp cell cultures that were corrected by the constitutive expression of the full-length human *DMP1* gene.^26^ The following study sought to identify the genetic pathways affected by the induction of odontogenic differentiation in XLH and XLH cells expressing *DMP1* in an effort to explain how *DMP1* contributed to enhanced matrix mineralization in our initial studies.

## Results

### Differentiation significantly upregulates inhibitors of the canonical Wnt pathway in XLH cells

**Fig. 1:**
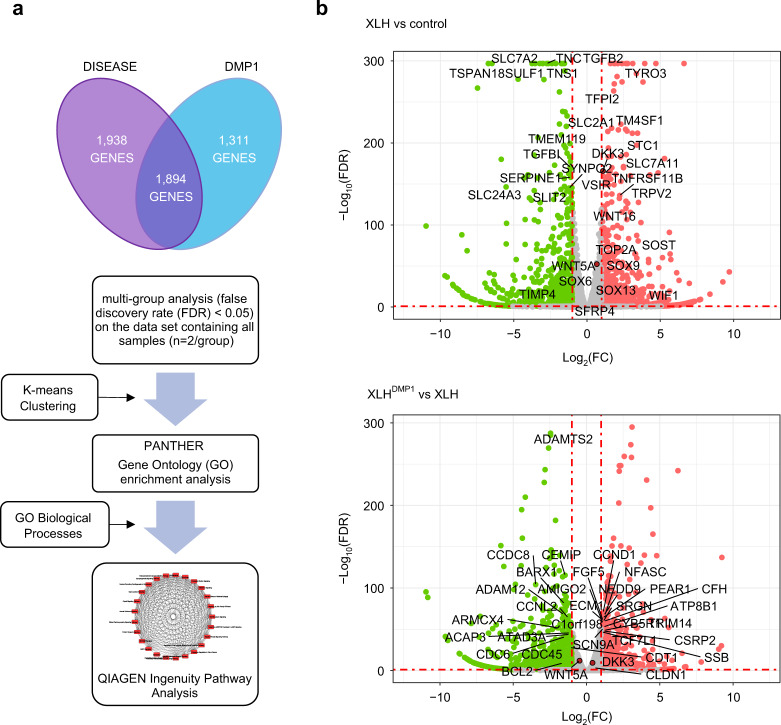
Genomic profiles of transgenic cells in response to differentiation. **a** ANOVA multi-group and multi-factor analysis were conducted on EdgeR to prioritize genes affected by disease and DMP1 status. Venn diagram represents transcripts with significant interaction and individual main effects combined (false discovery rate, FDR < 0.01). Disease status affected the expression of 3832 genes, while constitutive *DMP1* expression affected the expression of 3205 genes. *K*-means clustering, gene ontology, and pathway analyses were performed to identify interesting biological processes affected by disease and *DMP1* expression. **b** Volcano plots to present the distribution of differentially expressed genes. Dots in gray are those genes that did not meet the criteria of being significantly expressed with a twofold change or greater. Thresholds appear as red dashed lines on the *y*-axis for significance (FDR < 0.01), y-intercept at −Log10(FDR) = 2, and on the *x*-axis for fold-change (FC), x-intercepts at Log2(FC) = −1 and 1 (twofold decrease or increase, respectively). Dots in green denote downregulated genes, and dots in red denote upregulated genes

Transcription profiles of XLH dental pulp cells cultured for eight hours in differentiation media were analyzed. ANOVA multi-group and multi-factor analyses revealed that disease status affected the expression of 3832 genes, while constitutive *DMP1* expression affected the expression of 3205 genes (Fig. 1a). When compared to control (Ctrl) patients, XLH patients presented with significantly higher expression of sclerostin (*SOST*), WNT Inhibitory Factor 1 (*WIF1*), dickkopf 3 (*DKK3*) a Wnt signaling pathway inhibitor, and Wnt family members 5A and 16 (*WNT5A* and *WNT16*) (Fig. 1b).

K-means clustering (*k* = 9), gene ontology (GO), and pathway analyses were performed to identify interesting biological processes affected by disease and *DMP1* expression. Cluster 4 genes were significantly associated with GO terms of interest in odontoblast differentiation, namely collagen fibril organization (GO: 0030199), positive regulation of the Wnt signaling pathway (GO: 0030177), and angiogenesis (GO:0001525). The heatmap representing cluster 4 genes highlights regions where *DMP1* expression normalized gene expression (Supplementary Fig. 1).

### DMP1 reverses the expression of Wnt pathway inhibitors in XLH cells

**Fig. 2:**
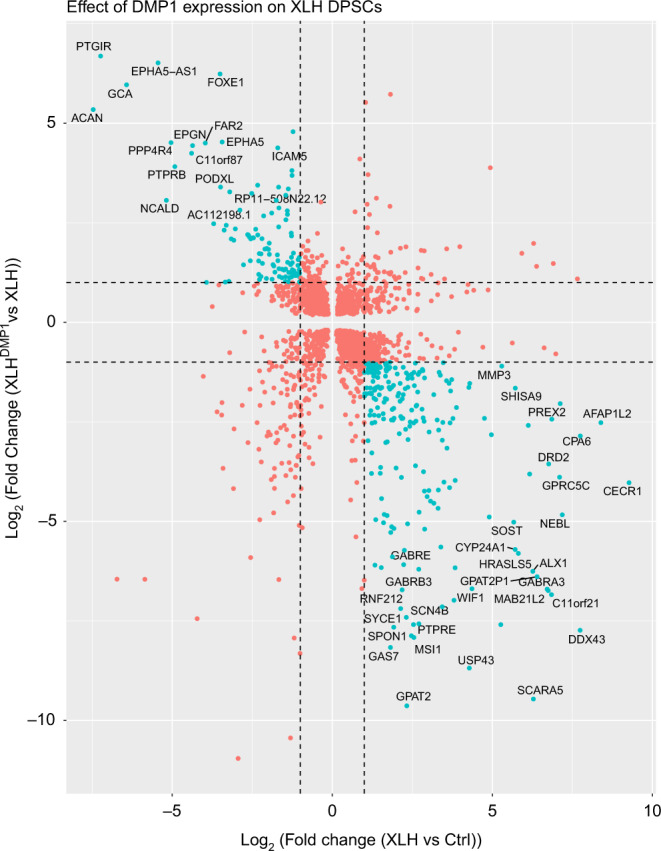
Effect of *DMP1* expression on XLH DPSCs. A post hoc pairwise comparison was conducted between the samples. First, significantly differentially expressed genes (DEGs)(FDR < 0.01) between Ctrl and XLH samples were identified. This list of genes was further restricted to those genes that were differentially expressed between XLH^DMP1^ and XLH samples. A total of 778 DEGs are plotted (red and blue dots). The dotted lines represent Log_2_(FC) = −1 and 1 threshold (twofold decrease or increase, respectively). In blue, 336 genes are highlighted which exhibited a reversal in expression pattern with *DMP1* expression (e.g., in the upper left quadrant are genes significantly downregulated in XLH vs. Ctrl cells, that were found to be upregulated in XLH^DMP1^ vs. XLH cells)

A post hoc pairwise comparison of differentially expressed genes (DEGs) expressing at least twofold changes between Ctrl, XLH, and XLH^DMP1^ was conducted. Out of the 778 DEGs, 336 genes exhibited a reversal in expression pattern and have been highlighted in blue (e.g., genes significantly downregulated in XLH were now found to be upregulated in XLH^DMP1^) (Fig. 2). The top DEGs have been labeled with their corresponding names. *WIF1* and *SOST* are among the highly expressed XLH genes whose expression declined upon *DMP1* expression.

**Fig. 3:**
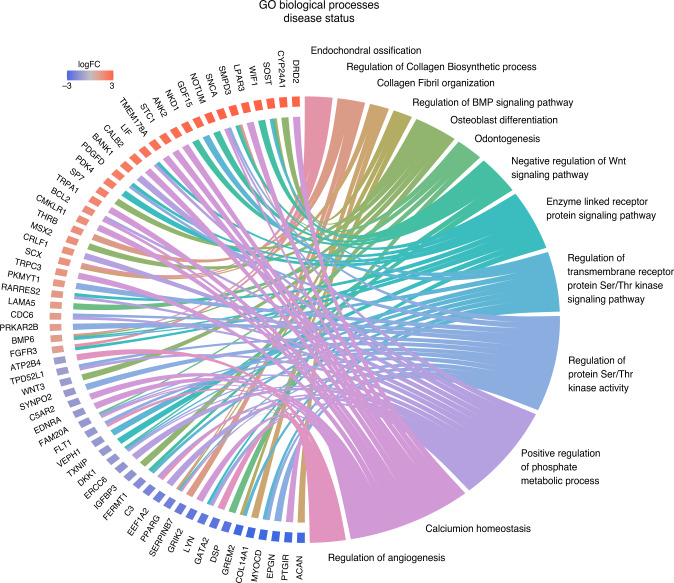
Top DEGs in XLH and Corresponding GO Terms. The list of 778 DEGs between Ctrl, XLH, and XLH^DMP1^ samples was submitted through GO enrichment analysis in PANTHER. The complete GO biological process annotation data set was used, including both manually curated and electronic annotations (GO Ontology database 10.5281/zenodo.5228828 Released 2021-08-18). A total of 748 had uniquely mapped IDs (30 gene IDs were unmapped, 16 were redundant and counted only once). The Fisher’s Exact Test with FDR correction, significance threshold set to FDR < 0.05. A subset of highly enriched GO Biological Processes was identified and further analyzed. These GO processes contained 180 unique genes. Their membership to each GO term and their differential expression in XLH vs. Ctrl cells are presented in this chord diagram. Only GO processes with at least three members are included

A total of 778 DEGs between XLH and Ctrl cells were uploaded to PANTHER for GO enrichment analysis. The chord diagram presents a subset of highly enriched GO Biological Processes, their constituent genes, and each gene’s corresponding expression pattern as log fold-change (Fig. 3). Among the enriched GO terms were those for collagen fibril organization (GO:0030199), osteoblast differentiation (GO:0001649), odontogenesis (GO:0042476), negative regulation of Wnt signaling pathway (GO:0030178), and regulation of angiogenesis (GO:0045765).

**Fig. 4:**
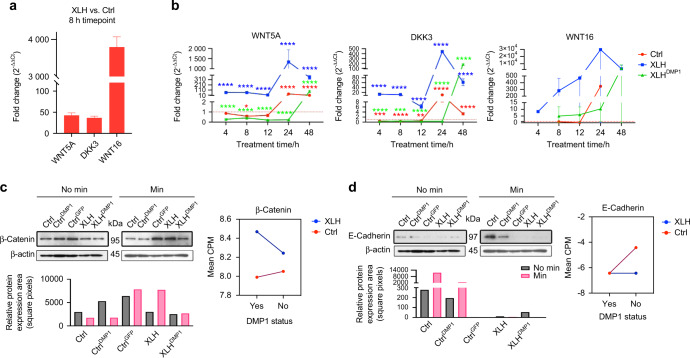
DMP1 reverses the expression of Wnt pathway Inhibitors in XLH cells. **a** Real-time PCR validation of RNA-seq data using a second biological sample, 8-h timepoint induction. XLH cells had significantly (*P* < 0.0001) higher levels of *WNT5A*, *DKK3*, and *WNT16* transcripts when compared to Ctrl cells. Fold-change in dCT values between XLH and Ctrl cells is presented. Two-way ANOVA, alpha = 0.05, with Sidak’s multiple comparison test. **b** Time series (0, 4, 8, 12, 24, and 48-h timepoints) of validated genes *WNT5A*, *DDK3*, and *WNT16*. Within the group, significance is denoted by asterisks of the corresponding color. The graph presents fold change (2^−ΔΔCT^) in gene expression; each timepoint was normalized to 0 h (except for XLH^DMP1^ *WNT16*, which was undetectable at 0 h and was normalized to 4 h). *WNT16* was not consistently expressed by all cell types across timepoints, therefore we were unable to report statistical significance. Two-way ANOVA, alpha = 0.05, with Tukey’s multiple comparisons. *P < 0.05, **P < 0.01, ***P < 0.001, ****P < 0.0001. **c**, **d** Representative Western blot showing analysis of E-cadherin and Beta-Catenin expression in response to differentiation, normalized to beta-actin loading control. **c** Under standard growth conditions (No Min, black bars), beta-catenin protein levels were higher in Ctrl^DMP1^ and Ctrl^GFP^ cells than in XLH and XLH^DMP1^ cells. Beta-catenin protein levels decreased with the induction of differentiation (Min, pink bars) in both Ctrl and Ctrl^DMP1^ cells, but increased in XLH and XLH^DMP1^ cells. Interaction plots representing the RNAseq multigroup analysis. The multigroup analysis revealed that beta-catenin expression differences between Ctrl and XLH cells (main effect *Q* = 1.23E−05) depended on *DMP1* status (interaction effect Q = 1.79E−03). Under Min conditions, Ctrl cells expressed lower transcript counts than XLH cells. This pattern was also observed in protein expression. *DMP1* transduction resulted in greater beta-catenin transcript levels in XLH cells and decreases in Ctrl cells. This pattern was not observed in Ctrl^DMP1^ and XLH^DMP1^ protein levels. **d** Under standard growth conditions (black bars), E-cadherin protein levels were highest in Ctrl cells. E-cadherin protein levels increased with the induction of differentiation (Min, pink bars) in both Ctrl and Ctrl^DMP1^ cells, but remained absent or decreased in the remaining cell types. Interaction plots representing the RNAseq multigroup analysis. The RNAseq multigroup analysis revealed significant individual main effects for Disease status (*Q* = 8.54E−03), not dependent on *DMP1* status or Phosphate source. We observe higher transcript counts in Ctrl cells when compared to XLH cells. E-cadherin gene expression did not differ between Ctrl^DMP1^ and XLH^DMP1^ cells, although protein levels were lower in XLH^DMP1^ cells. CPM, counts per million, in log2-scale, with a pseudo-count added to prevent taking the log of 0. Negative numbers indicate lower expression. Min, mineralization/differentiation conditions. No Min, standard growth conditions. Western blots for the second set of experiments can be found in Supplementary Materials

Real-time PCR was used to assess the expression pattern of the validated genes *WNT5A*, *DKK3*, and *WNT16* in response to DMP1 expression (Fig. 4a, b). Gene expression was determined at 0, 4, 8, 12, 24, and 48-h timepoints. *WNT5A* and *DKK3* gene expression increased significantly with time in XLH cells. *DMP1* expression in XLH^DMP1^ cells resulted in a decrease in both markers to levels comparable to Ctrl cells. No significant differences were observed between Ctrl and XLH *DKK3* levels at 0 h (*P* = 0.9998) or between Ctrl and XLH^DMP1^ *WNT5A* levels at 12 h (*P* = 0.0725). *WNT16* was not consistently expressed by all cell types across timepoints and was undetectable in Ctrl cells at 4- and 48-h timepoints and in XLH^DMP1^ cells at the 0-h timepoint (Fig. 4b). *WIF1* and *SOST* were undetectable at the 8-h time point using real-time PCR (data not shown). Further optimization of primers and PCR conditions is needed to validate these two important markers.

### Inhibition of E-cadherin and activation of beta-catenin in response to XLH differentiation

E-cadherin is one component of adherens junctions necessary for palisade formation that is transcriptionally regulated by the Wnt pathway but also sequesters beta-catenin limiting its downstream Wnt pathway functions.^20^ Beta-catenin protein levels decreased with the induction of differentiation (Min, pink bars) in both Ctrl and Ctrl^DMP1^ cells but increased in XLH and XLH^DMP1^ cells (Fig. 4c). E-cadherin protein levels increased with the induction of differentiation (Min, pink bars) in both Ctrl and Ctrl^DMP1^ cells but remained absent or decreased in the remaining cell types (Fig. 4d). Under standard growth conditions (No Min, black bars), protein levels were highest in Ctrl cells, and higher in Ctrl^DMP1^ and Ctrl^GFP^ cells than in XLH and XLH^DMP1^ cells. Corresponding interaction plots from the RNA-seq multigroup analysis revealed that beta-catenin expression differences between Ctrl and XLH cells (main effect *Q* = 1.23E−05) depended on *DMP1* status (interaction effect *Q* = 1.79E−03). Under odontogenic differentiation culture medium conditions, Ctrl cells expressed lower beta-catenin transcript counts than XLH cells. This pattern was also observed with the protein expression of beta-catenin. *DMP1* expression resulted in greater beta-catenin transcript levels in XLH cells and decreases in Ctrl cells. This pattern was not observed in Ctrl^DMP1^ and XLH^DMP1^ protein levels.

## Discussion

Transcriptomic analysis of XLH dental pulp cells has not been previously reported. The following study proposes a mechanism by which dentin formation and mineralization are affected in XLH individuals. That is, a defect in the Wnt signaling pathway responsible for odontogenic differentiation is present in the disease. XLH is an inherited metabolic disorder of fibroblast growth factor 23 (FGF23) excess that creates an antagonistic environment to bone formation. Such an environment would reasonably result in Wnt signaling pathway suppression, as this pathway is intractably associated with bone formation.^27^ Despite an extremely limited sample size and a lack of age-, sex-matching available, the similarities found in the Wnt profiles of these patients suggest disruptions independent of these parameters. Complete penetrance of the genotype without differences between males and females may explain this observation.^28^ Validation of the RNA sequencing data in the second XLH patient suggests that further study should follow to understand the effects of Phex dysfunction on the Wnt pathway Table 1.

**Table 1:** Real-time PCR primers for RNA sequencing validation

Target	Accession number	Forward primer sequence	Reverse primer sequence
GAPDH	NM_002046.7	ATCCCATCACCATCTTCCAG	GAGTCCTTCCACGATACCAA
ACTB	NM_001101	AAACTGGAACGGTGAAGGTG	AGAGAAGTGGGGTGGCTTTT
WNT5A	NM_003392	GCCAGTATCAATTCCGACATCG	TCACCGCGTATGTGAAGGC
DKK3	NM_013253	ATGTGTGCAAGCCGACCTT	CCTCAGCGCCATCTCTTCA
WNT16	NM_016087	GCAGAGAATGCAACCGTACAT	CACATGGGTGTTGTAACCTCG

We showed that XLH pulp cells upregulate inhibitors of the canonical Wnt pathway in response to the induction of odontogenic differentiation. These genes included *SOST*, *WIF1*, *WNT16*, *WNT5A*, and *DKK3*, the latter three of which have been validated (Table 2). Time course experiments revealed that *WNT16*, *WNT5A*, and *DKK3* expression was highest in XLH cells, peaking at 24-h (Fig. 4b). Despite this 24-h peak, which is also seen in Ctrl cells, it is important to note that sufficiently detectable differences in expression levels were observed at baseline and with *DMP1* expression in XLH cells. *DMP1* was able to suppress the transcription of these genes up until the 48-h timepoint, at which point expression returned to XLH levels. The return to baseline in XLH^DMP1^ cells may offer an explanation for the failure to rescue the XLH phenotype in vivo using *DMP1*.^26^ Future experiments should assess the time-dependent expression of these proteins relative to their unique roles in odontoblast differentiation. Despite increases in *WNT5A*, *WNT16*, and *DDK3*, the accumulation of beta-catenin in XLH cells in response to induction may suggest either faulty inhibition or communication between established pathways leading to canonical pathway activation (Fig. 4c). Future experiments should differentiate between nuclear and cytoplasmic, active and inactive, beta-catenin to better understand what was observed in XLH cells since only nuclear beta-catenin can mediate transcription.

**Table 2:** Negative regulators of the canonical Wnt signaling pathway

Gene name	XLH/Ctrl: logFC^a^	XLH/Ctrl: *Q* value^b^	XLH^DMP1^/XLH: logFC^a^	XLH^DMP1^/XLH: *Q* value^b^
SOST	5.67	2.07E−65	−5.02	2.19E−60
WIF1	4.36	3.79E−04	−6.69	4.13E−05
WNTl6	2.66	4.22E−121	0.33	4.01E−04
WNT5A	0.67	5.04E−53	−0.48	l.32E−12
DKK3	1.09	1.18E−166	0.41	5.30E−10

^a^Log_2_ Fold-change (e.g., 0 = no change, 2 = 4-fold increase, −2 = 4-fold decrease, etc). To reverse the order of the comparison, reverse the sign (+2 becomes −2; e.g., logFC is calculated as Disease/Control, but you want to see Control/Disease)

^b^Corrected *P*-value (i.e., false discovery rate)

Alternatively, a positive correlation between *WNT5A* activity, Notch signaling, and dental pulp stem cell differentiation suggests that other pathways may interconnect and play equally important roles.^29^ We have previously shown that calcium-binding proteins, such as DMP1, can activate the serine-threonine Ca^2+^/calmodulin-dependent protein kinase II (CaMKII) and mediate odontoblast differentiation.^30–32^ WNT5A has also been linked to Notch signaling activation via CaMKII activity.^33^ Calcium ion homeostasis, another putative biological process involved in XLH pathology (Fig. 3), along with its role in non-canonical Wnt signaling and pathways such as Notch signaling, must be considered in future studies.

Previous reports of small interfering RNA (siRNAs) silencing of the *PHEX* gene have revealed a subsequent downregulation of the Wnt pathway upon WNT3A stimulation. Furthermore, genome-wide RNA interference (RNAi) screens for Wnt/beta-catenin pathway components identified *PHEX* as a positive regulator of this pathway.^34,35^

Canonical Wnt signaling is important for the survival of undifferentiated dental pulp cells and promotes odontoblast differentiation and mineral formation, in vitro.^36^ Disruption of canonical Wnt signaling results in defects in dentin apposition, root and molar cusp development, and even tooth agenesis.^37,38^ WNT5A antagonizes canonical Wnt/beta‐catenin signaling and stimulates non-canonical WNT siganling.^39,40^ Elevated levels of other canonical Wnt pathway inhibitors, namely sclerostin (SOST), have been identified in XLH patients.^41^ Immunotherapies neutralizing sclerostin activity have, in fact, proven successful in improving bone mass, formation rate, and strength in Hyp mice.^42,43^ The effects of suppressing these Wnt signaling inhibitors in the tooth may also prove a useful model for understanding the pathophysiology of XLH.

Despite reports of downregulation of canonical Wnt pathway inhibitors, DKK1, sFRP-2, sFRP-4, and WIF1, during osteoblastic differentiation, absolute depletion of sFRP2 has been associated with the inhibition of odontogenic differentiation in mesenchymal stem cells.^44,45^ The upregulation of sFRP-2 was observed during odontogenic differentiation of stem cells of the apical papilla, resulting in increased *DMP1* gene expression among other markers of differentiation.^45^

In fact, studies in periodontal ligament stem cells have shown that inhibition of Wnt signaling is required for the maintenance of the osteogenic potential of these cells.^46^ Meanwhile, increased Wnt signaling, such as in klotho-deficient mice, results in accelerated cellular senescence.^47^ Furthermore, constitutive activation of Wnt signaling, such as in NOTUM knockout mice, manifests as dentin dysplasia, periodontal inflammation, and periapical abscess formation.^6,8–10^ These studies highlight the need for further investigation of the temporal regulation of these pathways. The significance of our observations would likely lie within the context of temporal regulation.

Cadherins play a role in the cell–cell junctions of epithelial cells. In vivo studies have shown that differentiating odontoblasts express high levels of N-cadherin and no E-cadherin, while functional odontoblasts express low levels of E-cadherin and high levels of N-cadherin.^48^ In vitro studies, on the other hand, have shown that induction with 10 mM beta-glycerophosphate results in a gradual increase in E-cadherin and a gradual decrease in N-cadherin.^49^ Our Ctrl cells corroborate the latter findings better than the former.

E-cadherin is associated with the polarized epithelial phenotype. E-cadherin protein levels were highest in Ctrl cells under standard conditions (No Min) and increased further with the induction of differentiation in both Ctrl and Ctrl^DMP1^ cells. By contrast, a decrease in E-cadherin was observed in XLH and XLH^DMP1^ cells with the induction of differentiation. This pattern was observed in transcript numbers, as well. Through RNA-seq, we find that N-cadherin (*CDH2*) is also downregulated in XLH cells. Given the poorly polarized, disorganized odontoblast layer in XLH teeth, it is possible that the observed reduction in E-cadherin may be affecting XLH cell odontoblast layer formation.^7^ While E-cadherin decreases in XLH and XLH^DMP1^ cells with differentiation, beta-catenin protein levels increased in XLH and XLH^DMP1^ cells when compared to standard growth conditions. By contrast, beta-catenin protein levels decreased after induction in Ctrl and Ctrl^DMP1^ cells, concurrent with the observed increase in E-Cadherin protein. These changes could have downstream effects on cell attachment, Wnt signaling, and cell differentiation.

**Fig. 5:**
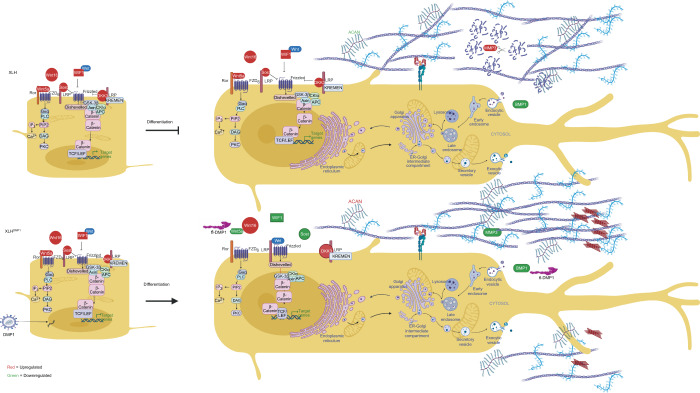
Constitutive expression of DMP1 promotes canonical Wnt signaling. XLH dental pulp cells exhibited impaired differentiation due to the upregulation of inhibitors of the canonical Wnt pathway, such as *WNT5a,* *WNT16,* *WIF1,* and *SOST.* Constitutive expression of full-length DMP1 (fl-DMP1) resulted in the downregulation of these Wnt inhibitors, restoring differentiation potential. Constitutive DMP1 expression in XLH dental pulp cells resulted in improved mineralization. BMP1 bone morphogenetic protein 1, DKK3 Dickkopf-related protein 3, MMP3 matrix metalloproteinase 3, WIF1 Wnt inhibitory factor 1, SOST sclerostin. Created with BioRender.com

Induction of odontogenic differentiation resulted in the upregulation of inhibitors of the canonical Wnt pathway in XLH cells, while constitutive expression of full-length DMP1 in XLH cells reversed this effect (Fig. 5). The question that arises is that of DMP1’s role in restoring Wnt signaling in these cells. The answer to this question may be more challenging than we would like. The markers implicated in this disease, namely FGF23, Vitamin D, parathyroid hormone, and the sodium–phosphate co-transporters, are all part of a bigger network for calcium and phosphate metabolism. Defining PHEX function and its interaction with DMP1 would thus require a thorough understanding of the physiology of mineral metabolism and its relationship to Wnt signaling.

## Materials and methods

### Cell culture

**Fig. 6:**
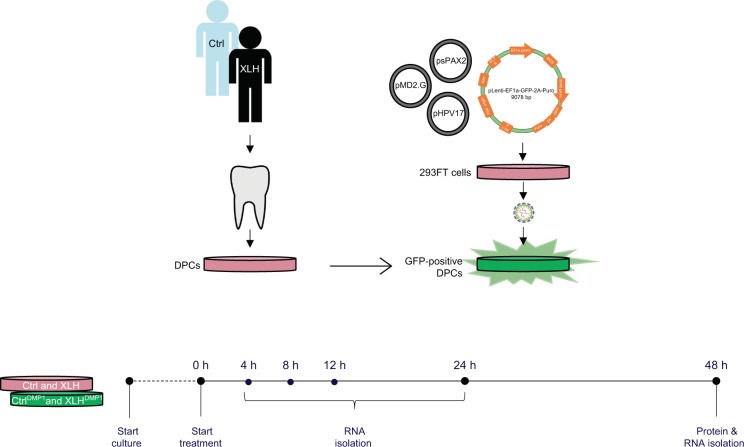
Experimental design. Dental pulp cells were isolated from the third molars of XLH and healthy controls. The calcium phosphate transfection method was used to transfect full-length human *DMP1* cDNA into low-passage 293FT cells using a lentivirus plasmid (pLenti-DMP1-GFP-2A-Puro), together with the psPAX2 (Addgene), pMD2.G (Addgene), and pHPV17 plasmids. Stable transduction of the full-length human *DMP1* gene was achieved by producing control (Ctrl) and XLH cells overexpressing *DMP1* (Ctrl^DMP1^ and XLH^DMP1^)(Guirado et al., 2020). Odontogenic differentiation of the cells was performed, and RNA was isolated at 4, 8, 12, 24, and 48 h of culture. Eight-hour samples were chosen for RNA sequencing as this was the earliest time point at which gene expression changes were observed. Protein was isolated at the 48-h timepoint

Dental pulp cells were isolated from the third molars of XLH and healthy controls (*N* = 2 per genotype) and stable transduction of full-length human *DMP1* gene was achieved, as previously described, producing control (Ctrl) and XLH cells overexpressing *DMP1* (Ctrl^DMP1^ and XLH^DMP1^).^26^ Empty vectors were transduced as controls, producing Ctrl^GFP^ cells. Cells (under seven passages) were plated at a density of 3.125 × 10^4^ cells per cm^2^ and cultured in odontogenic differentiation media (Dulbecco’s Modified Eagle Medium 1 g·L^−1^ d-Glucose (DMEM; Invitrogen, Grand Island, NY, USA) supplemented with 10% fetal bovine serum (Invitrogen), 1% antibiotic–antimycotic 100× (Gibco/Invitrogen, Cat. 15240062), ascorbic acid (0.50 mmol·L^−1^), β-glycerophosphate (10 mM), and dexamethasone (10 nmol·L^−1^)) for 8 h, 37 °C, 5% CO_2_. Conditions were repeated in duplicates. After 8 h, RNA was isolated with the miRNeasy Mini Kit (Cat. No. 217004). No DNase treatment was performed. One microgram of RNA was submitted to the RNA-sequencing core facility. Real-time PCR validation of RNA-sequencing data was performed using a second patient sample. Eight hours was the earliest timepoint at which gene expression changes occurred, per our preliminary studies. Where applicable, a series of collection timepoints were used to evaluate changes in gene expression over time (Fig. 6).

### Protein isolation and Western blot analyses

Cell pellets were resuspended in 500 μl RIPA buffer (10× RIPA buffer with protease inhibitors). Lysates were incubated on a shaker for one hour at 4 °C, after which they were centrifuged for 30 min at 19 467 × *g* to remove cell debris. Supernatant protein concentration was assessed using the Bradford assay, 20 μg total protein was resolved using a 10% SDS-PAGE gel at 180 V for 50 min and then transferred onto PVDF membranes at 22 V overnight. Membranes were blocked in 5% dried milk in phosphate-buffered saline (PBS). Primary antibodies against beta-catenin (Sigma-Aldrich No. 04-958; dilution 1:1 000) and E-cadherin (Santa Cruz No. sc-8426; dilution 1:200), and HRP-conjugated secondary antibodies were resuspended at the appropriate concentrations in 5% dried milk in PBS. Western blot was developed using Pierce enhanced chemiluminescence (ECL) Plus western blotting substrate (ThermoFisher, Cat. No. 32106). Western blot analysis was performed on ImageJ.^50^ Scanned western blot film images were uploaded to ImageJ, image type was changed to 8-bit to allow for light background subtraction. Lanes were plotted using the Gel Analysis Tool, and the area under the curve was calculated. Standardization of each lane was done accordingly to their corresponding loading control (β-actin). The second experiment can be found in supplementary materials.

### RNA sequencing quality control and quantification

RNA sequencing was conducted at the UIC Research Resources Center (GEO accession GSE201313). RNA integrity was assessed using Agilent TapeStation 4200 (all samples had RIN scores above nine). Library construction was based on Universal Plus mRNA-seq chemistry by NuGEN. Sequencing was performed on the NovaSeq 6000 instrument with SP flow cell (2 × 50 reads), 380+ million reads per lane, and approximately 23 million clusters/sample.

Raw sequencing reads were aligned to the human reference genome (HG38) using the STAR aligner and ENSEMBL gene and transcript annotations.^51^ Gene expression levels were quantified using FeatureCounts^52^ as raw read counts and as normalized reads-per-million. Normalized expression (in counts per million) accounts for differences in sequencing depth across libraries, allowing expression levels to be directly compared between samples.

Quality control was performed to confirm the depth and quality of the raw sequencing data and the absence of sequencing artifacts and to confirm that the number of reads aligning to the reference genome mapping to coding sequences was sufficient for expression estimates. Prior to differential expression analysis, principal component analysis (PCA) was performed to identify biological outliers that should be removed or further investigated. PCA plots and RNA integrity information can be found in Supplementary Materials.

### Bioinformatics analysis

Additional normalization with TMM (trimmed mean of M-values) scaling was performed in edgeR. TMM normalization is more robust to outlier features and seeks to ensure that the average log-fold change across samples is 0. Pseudo-counts were added to prevent taking the log of 0. Negative numbers simply indicate lower expression. Differential expression statistics (fold-change and p-value) were computed from raw expression counts using edgeR.^53,54^ Multi-group and multi-factor analyses and post-hoc pairwise analyses were performed. The false discovery rate (FDR) correction of Benjamini and Hochberg was used to correct for multiple comparisons.^55^ Significant genes were determined based on an FDR threshold of 5% (0.05) in the multi-group comparison.

### GO analysis

GO enrichment analysis was conducted on PANTHER.^56^ Analysis type utilized PANTHER Overrepresentation Test (Released 20210224). The complete GO biological process annotation data set was used, including both manually curated and electronic annotations (GO Ontology database 10.5281/zenodo.5228828 Released 2021-08-18). All *Homo sapiens* genes in the database were used as the reference list.

The Fisher’s exact test with FDR correction (FDR-adjusted *P*-value < 0.05) was used to identify the top three significantly enriched GO biological processes. Fold enrichment is presented as the number of genes in the cluster divided by the expected number of genes based on the reference list. Fold enrichment greater than one indicates that the GO term is overrepresented in the cluster.

### Pathway analyses

Qiagen Ingenuity Pathway Analysis software was utilized.^57^ Pairwise comparisons were matched to the Ingenuity Pathway Analysis library of canonical pathways. A Fisher’s Exact test (alpha = 0.01) was performed, generating a −log(*P*-value), and a cutoff of 2 was chosen. DEGs from our data (FDR < 0.01) that were associated with a canonical pathway in the Ingenuity Knowledge Base were considered for the analysis.

## Supplementary information

RNA Integrity

Principal Component Analysis

Supplementary Figure 1

## Data Availability

All RNA sequencing data is available via Gene Expression Omnibus, GEO accession GSE201313. Additional data may be made available upon request.

## References

[1] Beck-Nielsen SS, Brock-Jacobsen B, Gram J, Brixen K, Jensen TK (2009). Incidence and prevalence of nutritional and hereditary rickets in southern Denmark. Eur. J. Endocrinol..

[2] Endo I (2015). Nationwide survey of fibroblast growth factor 23 (FGF23)-related hypophosphatemic diseases in Japan: prevalence, biochemical data and treatment. Endocr. J..

[3] Rafaelsen S, Johansson S, Ræder H, Bjerknes R (2016). Hereditary hypophosphatemia in Norway: a retrospective population-based study of genotypes, phenotypes, and treatment complications. Eur. J. Endocrinol..

[4] Foster BL (2014). Rare bone diseases and their dental, oral, and craniofacial manifestations. J. Dent. Res..

[5] Foster BL, Nociti FH, Somerman MJ (2014). The rachitic tooth. Endocr. Rev..

[6] Hanisch M, Bohner L, Sabandal MMI, Kleinheinz J, Jung S (2019). Oral symptoms and oral health-related quality of life of individuals with x-linked hypophosphatemia. Head. Face Med..

[7] Salmon B (2014). Abnormal osteopontin and matrix extracellular phosphoglycoprotein localization, and odontoblast differentiation, in X-linked hypophosphatemic teeth. Connect. Tissue Res..

[8] Baroncelli GI (2006). Prevalence and pathogenesis of dental and periodontal lesions in children with X-linked hypophosphatemic rickets. Eur. J. Paediatr. Dent..

[9] Baroncelli GI (2021). Pulp chamber features, prevalence of abscesses, disease severity, and PHEX mutation in X-linked hypophosphatemic rickets. J. Bone Miner. Metab..

[10] Lo SH, Lachmann R, Williams A, Piglowska N, Lloyd AJ (2020). Exploring the burden of X-linked hypophosphatemia: a European multi-country qualitative study. Qual. Life Res..

[11] Salmon B (2013). MEPE-derived ASARM peptide inhibits odontogenic differentiation of dental pulp stem cells and impairs mineralization in tooth models of X-linked hypophosphatemia. PLoS ONE.

[12] Coyac BR (2017). Tissue-specific mineralization defects in the periodontium of the Hyp mouse model of X-linked hypophosphatemia. Bone.

[13] Hoshino M (2008). Claudin rather than occludin is essential for differentiation in rat incisor odontoblasts. Oral. Dis..

[14] Järvinen, E., Shimomura-Kuroki, J., Balic, A., Jussila, M. & Thesleff, I. Mesenchymal Wnt/β-catenin signaling limits tooth number. *Development* **145**, dev158048 (2018). 10.1242/dev.158048 29437780

[15] Zhao Y, Yuan X, Bellido T, Helms JA (2019). A correlation between Wnt/Beta-catenin signaling and the rate of dentin secretion. J. Endod..

[16] Zhu X (2013). Intra-epithelial requirement of canonical Wnt signaling for tooth morphogenesis *. J. Biol. Chem..

[17] Kim T-H (2011). Constitutive stabilization of ß-catenin in the dental mesenchyme leads to excessive dentin and cementum formation. Biochem. Biophys. Res. Commun..

[18] Zhao Y, Yuan X, Liu B, Tulu US, Helms JA (2018). Wnt-responsive odontoblasts secrete new dentin after superficial tooth injury. J. Dent. Res..

[19] Palacios J (1995). Differential spatiotemporal expression of E- and P-cadherin during mouse tooth development. Int. J. Dev. Biol..

[20] Hermans F, Hemeryck L, Lambrichts I, Bronckaers A, Vankelecom H (2021). Intertwined signaling pathways governing tooth development: a give-and-take between canonical Wnt and Shh. Front. Cell Dev. Biol..

[21] Kornsuthisopon C, Photichailert S, Nowwarote N, Tompkins KA, Osathanon T (2022). Wnt signaling in dental pulp homeostasis and dentin regeneration. Arch. Oral. Biol..

[22] Martin A (2011). Bone proteins PHEX and DMP1 regulate fibroblastic growth factor Fgf23 expression in osteocytes through a common pathway involving FGF receptor (FGFR) signaling. FASEB J..

[23] Lu Y (2011). The biological function of DMP-1 in osteocyte maturation is mediated by Its 57-kDa C-terminal fragment. J. Bone Miner. Res..

[24] Simsek Kiper PO (2016). Cortical-bone fragility—insights from sFRP4 deficiency in Pyle’s disease. N. Engl. J. Med..

[25] Palomo T, Glorieux FH, Rauch F (2014). Circulating sclerostin in children and young adults with heritable bone disorders. J. Clin. Endocrinol. Metab..

[26] Guirado E (2020). Disrupted protein expression and altered proteolytic events in hypophosphatemic dentin can be rescued by dentin matrix protein 1. Front. Physiol.

[27] Baron R, Kneissel M (2013). WNT signaling in bone homeostasis and disease: from human mutations to treatments. Nat. Med..

[28] Ruppe, M. D. X-Linked Hypophosphatemia. in GeneReviews(®) (eds Adam, M. P. et al.) (University of Washington, Seattle, 1993). 22319799

[29] Kornsuthisopon C (2022). Non-canonical Wnt signaling participates in Jagged1-induced osteo/odontogenic differentiation in human dental pulp stem cells. Sci. Rep..

[30] Eapen A (2010). Calcium-mediated stress kinase activation by DMP1 promotes osteoblast differentiation*. J. Biol. Chem..

[31] Eapen A (2013). Dentin phosphophoryn activates smad protein signaling through Ca2+-calmodulin-dependent protein kinase II in undifferentiated mesenchymal cells. J. Biol. Chem..

[32] Narayanan K (2003). Dual functional roles of dentin matrix protein 1 implications in biomineralization and gene transcription by activation of intracellular Ca2+ store. J. Biol. Chem..

[33] Ann E-J (2012). Wnt5a controls Notch1 signaling through CaMKII-mediated degradation of the SMRT corepressor protein. J. Biol. Chem..

[34] Tang W (2008). A genome-wide RNAi screen for Wnt/beta-catenin pathway components identifies unexpected roles for TCF transcription factors in cancer. Proc. Natl Acad. Sci. USA.

[35] Schmidt EE (2013). GenomeRNAi: a database for cell-based and in vivo RNAi phenotypes, 2013 update. Nucleic Acids Res..

[36] Vijaykumar A, Root SH, Mina M (2021). Wnt/β-catenin signaling promotes the formation of preodontoblasts in vitro. J. Dent. Res..

[37] Xu M (2017). WNT10A mutation causes ectodermal dysplasia by impairing progenitor cell proliferation and KLF4-mediated differentiation. Nat. Commun..

[38] Bae CH (2015). Wntless regulates dentin apposition and root elongation in the mandibular molar. J. Dent. Res..

[39] Kikuchi A, Yamamoto H, Sato A, Matsumoto S (2012). Wnt5a: its signalling, functions and implication in diseases. Acta Physiol..

[40] Oishi I (2003). The receptor tyrosine kinase Ror2 is involved in non-canonical Wnt5a/JNK signalling pathway. Genes Cells Devoted Mol. Cell. Mech..

[41] Ni X (2022). Low levels of serum sclerostin in adult patients with tumor-induced osteomalacia compared with X-linked hypophosphatemia. J. Clin. Endocrinol. Metab..

[42] Carpenter KA (2022). Sclerostin antibody improves phosphate metabolism hormones, bone formation rates, and bone mass in adult Hyp mice. Bone.

[43] Carpenter KA, Ross RD (2019). Sclerostin antibody treatment increases bone mass and normalizes circulating phosphate levels in growing Hyp mice. J. Bone Miner. Res..

[44] Mashhadikhan M, Kheiri H, Dehghanifard A (2020). DNA methylation and gene expression of sFRP2, sFRP4, Dkk 1, and Wif1 during osteoblastic differentiation of bone marrow derived mesenchymal stem cells. J. Oral. Biosci..

[45] Yu G (2016). Demethylation of SFRP2 by histone demethylase KDM2A regulated osteo-/dentinogenic differentiation of stem cells of the apical papilla. Cell Prolif..

[46] Liu H (2007). Augmented Wnt signaling in a mammalian model of accelerated aging. Science.

[47] Liu Q (2015). DKK1 rescues osteogenic differentiation of mesenchymal stem cells isolated from periodontal ligaments of patients with diabetes mellitus induced periodontitis. Sci. Rep..

[48] Heymann R (2002). E- and N-cadherin distribution in developing and functional human teeth under normal and pathological conditions. Am. J. Pathol..

[49] Lee H-K (2014). Nuclear factor I-C (NFIC) regulates dentin sialophosphoprotein (DSPP) and E-cadherin via control of Krüppel-like factor 4 (KLF4) during dentinogenesis*. J. Biol. Chem..

[50] Schneider CA, Rasband WS, Eliceiri KW (2012). NIH Image to ImageJ: 25 years of image analysis. Nat. Methods.

[51] Dobin A (2013). STAR: ultrafast universal RNA-seq aligner. Bioinformatics.

[52] Liao Y, Smyth GK, Shi W (2014). featureCounts: an efficient general purpose program for assigning sequence reads to genomic features. Bioinformatics.

[53] McCarthy DJ, Chen Y, Smyth GK (2012). Differential expression analysis of multifactor RNA-Seq experiments with respect to biological variation. Nucleic Acids Res..

[54] Robinson MD, McCarthy DJ, Smyth GK (2010). edgeR: a Bioconductor package for differential expression analysis of digital gene expression data. Bioinformatics.

[55] Benjamini Y, Hochberg Y (1995). Controlling the false discovery rate: a practical and powerful approach to multiple testing. J. R. Stat. Soc. Ser. B Methodol..

[56] Thomas PD (2003). PANTHER: a library of protein families and subfamilies indexed by function. Genome Res..

[57] Krämer A, Green J, Pollard J, Tugendreich S (2014). Causal analysis approaches in Ingenuity Pathway Analysis. Bioinformatics.

