# Supplementary material for: Wnt pathway inhibitors are upregulated in XLH dental pulp cells in response to odontogenic differentiation
Source: Int J Oral Sci. 2023 Feb 27;15:13. doi: 10.1038/s41368-022-00214-z (PMC9971210; doi:10.1038/s41368-022-00214-z)
Supplement: Supplementary file 1 — RNA Integrity [file 41368_2022_214_MOESM1_ESM.pdf]

Filename: GEO071619_RNA_dil1in5_072919.HSRNA

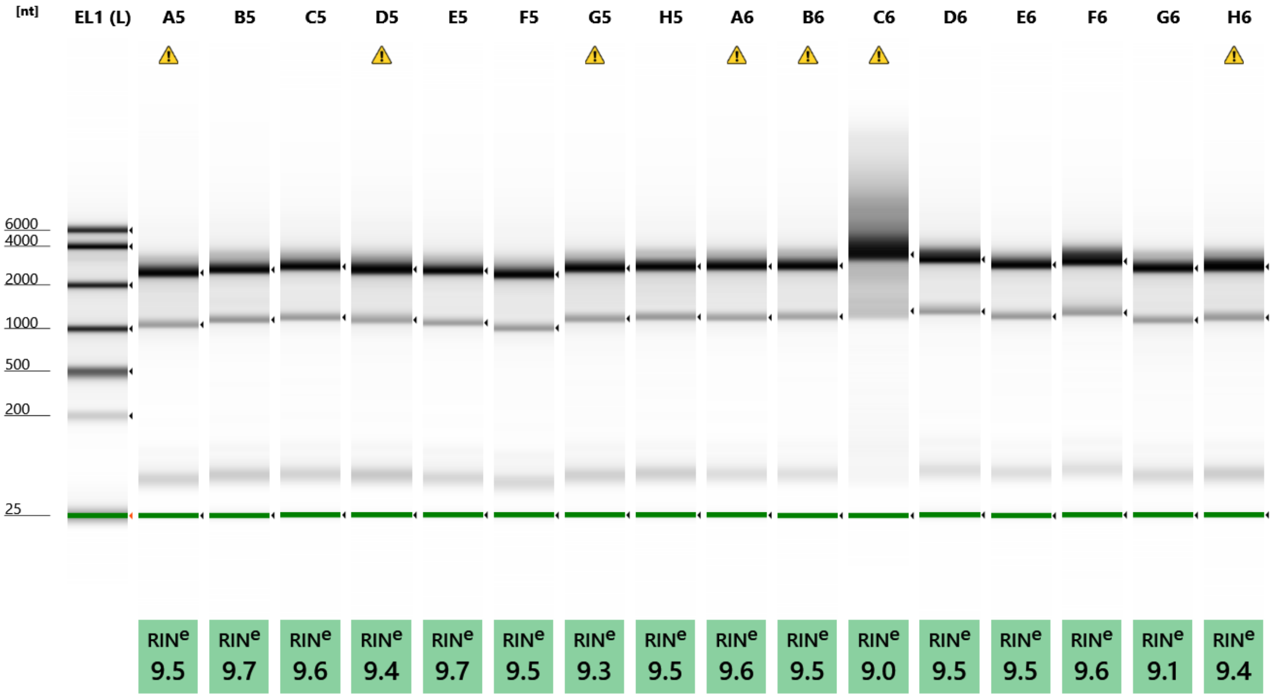

Default image (Contrast 50%), Image is Scaled to Sample

Sample Info

Well	RINe	28S/18S (Area)	Conc. [pg/µl]	Sample Description	Alert	Observations
EL1	-	-	3750	Electronic Ladder		Ladder
A5	9.5	3.8	33700	WT B 1	⚠	RNA concentration outside recommended range for RINe
B5	9.7	3.1	14800	WT B 2		
C5	9.6	3.7	19500	WT PI 1		
D5	9.4	3.5	49300	WT PI 2	⚠	RNA concentration outside recommended range for RINe
E5	9.7	3.9	23700	WTOE B 1		
F5	9.5	3.7	20000	WTOE B 2		
G5	9.3	3.5	32700	WTOE PI 1	⚠	RNA concentration outside recommended range for RINe
H5	9.5	3.0	22400	WTOE PI 2		
A6	9.6	4.0	25900	HYP B 1	⚠	RNA concentration outside recommended range for RINe
B6	9.5	3.4	37400	HYP B 2	⚠	RNA concentration outside recommended range for RINe
C6	9.0	7.9	305000	HYP PI 1	⚠	RNA concentration outside recommended range for RINe; RINe edited
D6	9.5	2.0	16300	HYP PI 2		
E6	9.5	3.4	18800	HYPOE B 1		
F6	9.6	1.7	20400	HYPOE B 2		
G6	9.1	3.2	20000	HYPOE PI 1		
H6	9.4	3.1	28800	HYPOE PI 2	⚠	RNA concentration outside recommended range for RINe

 © Agilent Technologies, Inc. 2017

EL1: Electronic Ladder

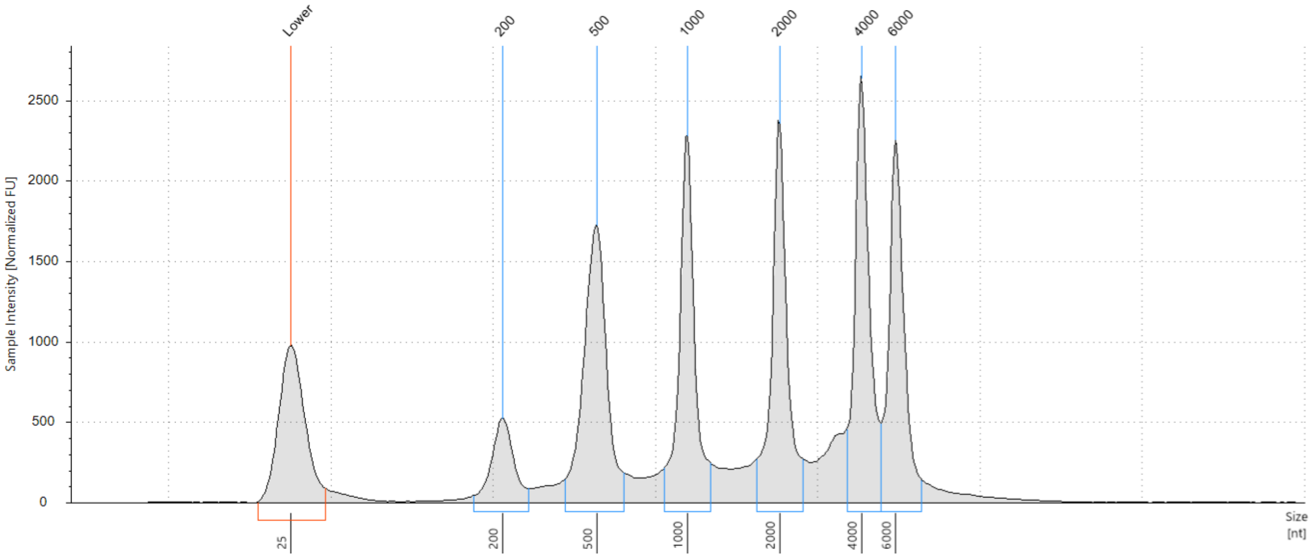

Sample Table

Well	RINe	28S/18S (Area)	Conc. [pg/µl]	Sample Description	Alert	Observations
EL1	-	-	3750	Electronic Ladder		Ladder

Peak Table

Size [nt]	Calibrated Conc. [pg/µl]	Assigned Conc. [pg/µl]	Peak Molarity [pmol/l]	% Integrated Area	Peak Comment	Observations
25	700	700	82400	-		Lower Marker
200	189	-	2770	5.98		
500	638	-	3760	20.25		
1000	557	-	1640	17.66		
2000	580	-	854	18.42		
4000	634	-	466	20.11		
6000	554	-	272	17.58		

 © Agilent Technologies, Inc. 2017

A5: WT B 1

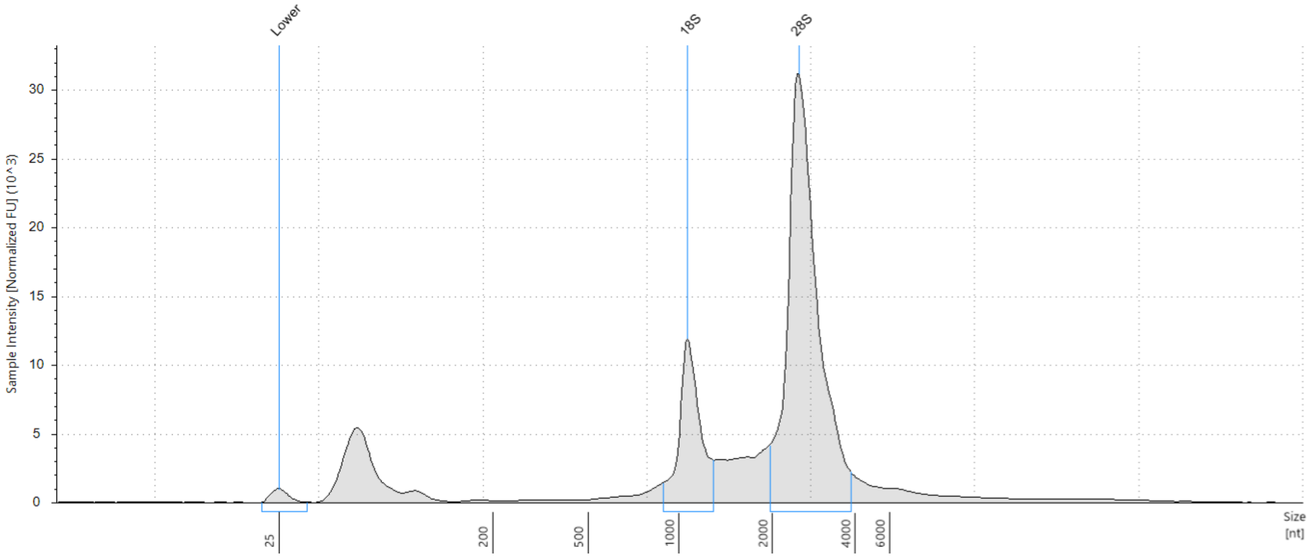

Sample Table

Well	RINe	28S/18S (Area)	Conc. [pg/µl]	Sample Description	Alert	Observations
A5	9.5	3.8	33700	WT B 1	⚠	RNA concentration outside recommended range for RINe

Peak Table

Size [nt]	Calibrated Conc. [pg/µl]	Assigned Conc. [pg/µl]	Peak Molarity [pmol/l]	% Integrated Area	Peak Comment	Observations
25	700	700	82400	-		Lower Marker
1064	4820	-	13300	20.78		18S
2493	18400	-	21700	79.22		28S

B5: WT B 2

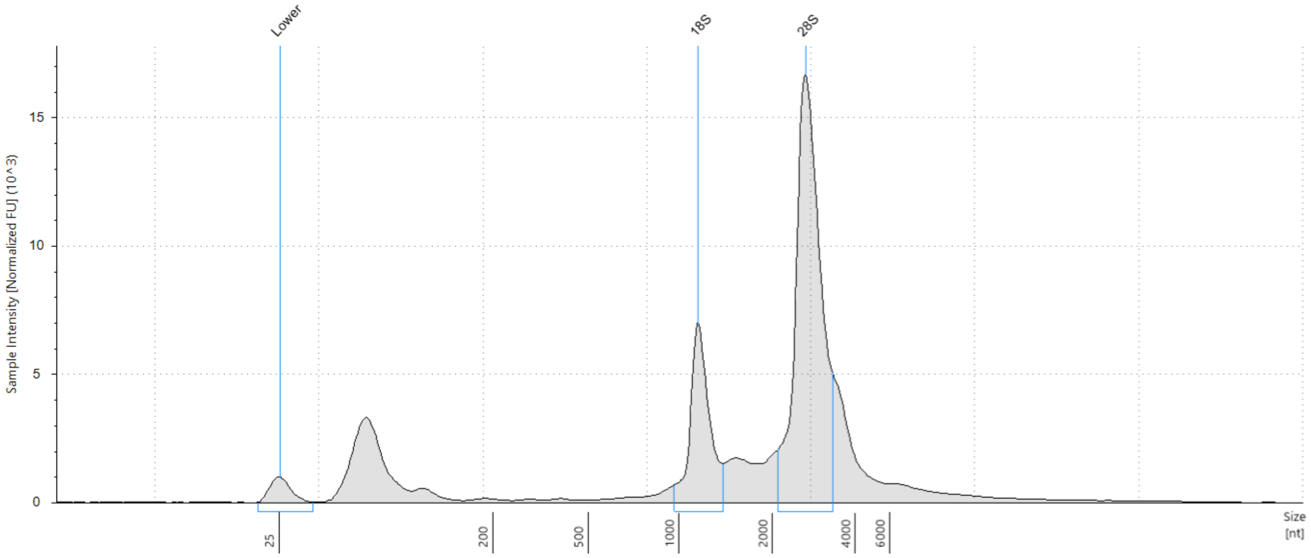

Sample Table

Well	RINe	28S/18S (Area)	Conc. [pg/µl]	Sample Description	Alert	Observations
B5	9.7	3.1	14800	WT B 2		

Peak Table

Size [nt]	Calibrated Conc. [pg/µl]	Assigned Conc. [pg/µl]	Peak Molarity [pmol/l]	% Integrated Area	Peak Comment	Observations
25	700	700	82400	-		Lower Marker
1145	2130	-	5460	24.19		18S
2622	6670	-	7480	75.81		28S

C5: WT PI 1

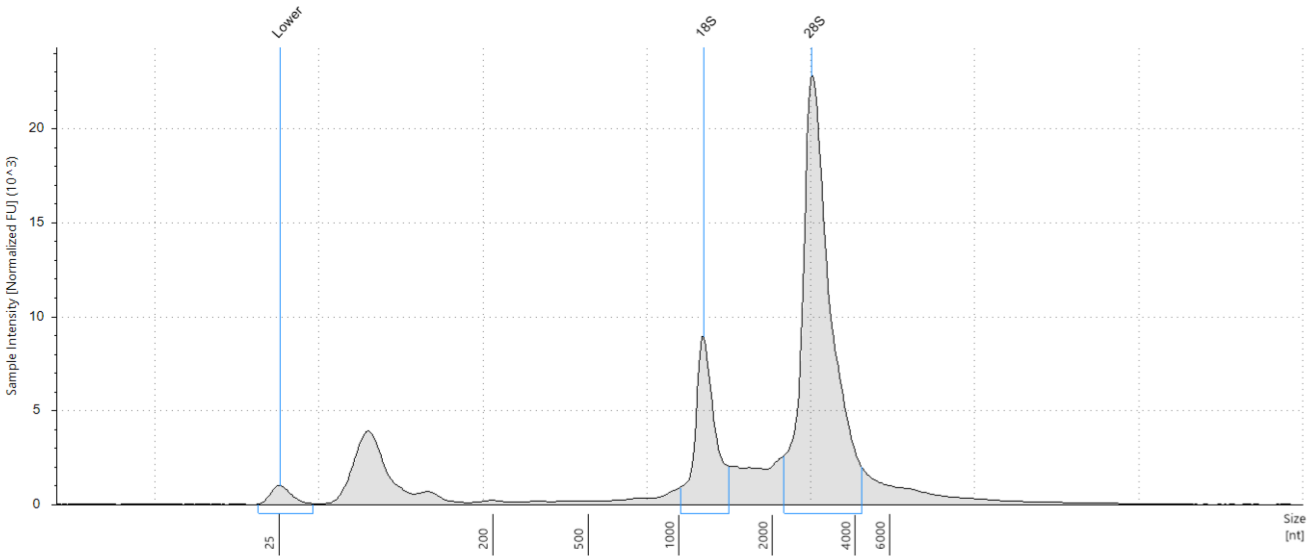

Sample Table

Well	RINe	28S/18S (Area)	Conc. [pg/µl]	Sample Description	Alert	Observations
C5	9.6	3.7	19500	WT PI 1		

Peak Table

Size [nt]	Calibrated Conc. [pg/µl]	Assigned Conc. [pg/µl]	Peak Molarity [pmol/l]	% Integrated Area	Peak Comment	Observations
25	700	700	82400	-		Lower Marker
1187	2830	-	7010	21.06		18S
2755	10600	-	11300	78.94		28S

© Agilent Technologies, Inc. 2017

D5: WT PI 2

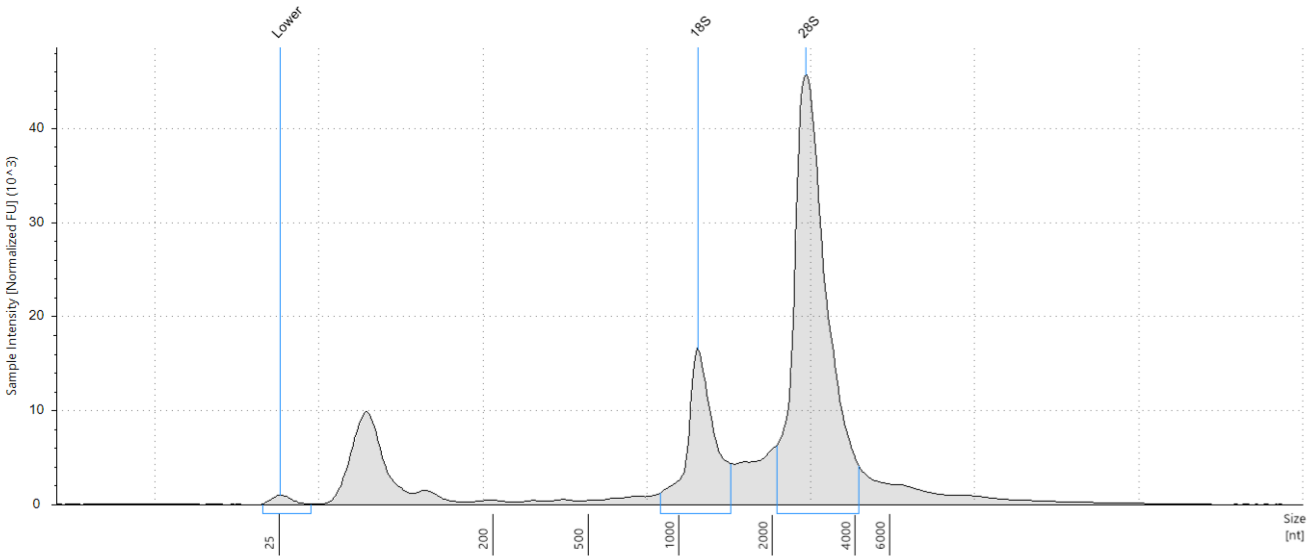

Sample Table

Well	RINe	28S/18S (Area)	Conc. [pg/µl]	Sample Description	Alert	Observations
D5	9.4	3.5	49300	WT PI 2	⚠	RNA concentration outside recommended range for RINe

Peak Table

Size [nt]	Calibrated Conc. [pg/µl]	Assigned Conc. [pg/µl]	Peak Molarity [pmol/l]	% Integrated Area	Peak Comment	Observations
25	700	700	82400	-		Lower Marker
1140	7540	-	19400	22.03		18S
2630	26700	-	29800	77.97		28S

 © Agilent Technologies, Inc. 2017

E5: WTOE B 1

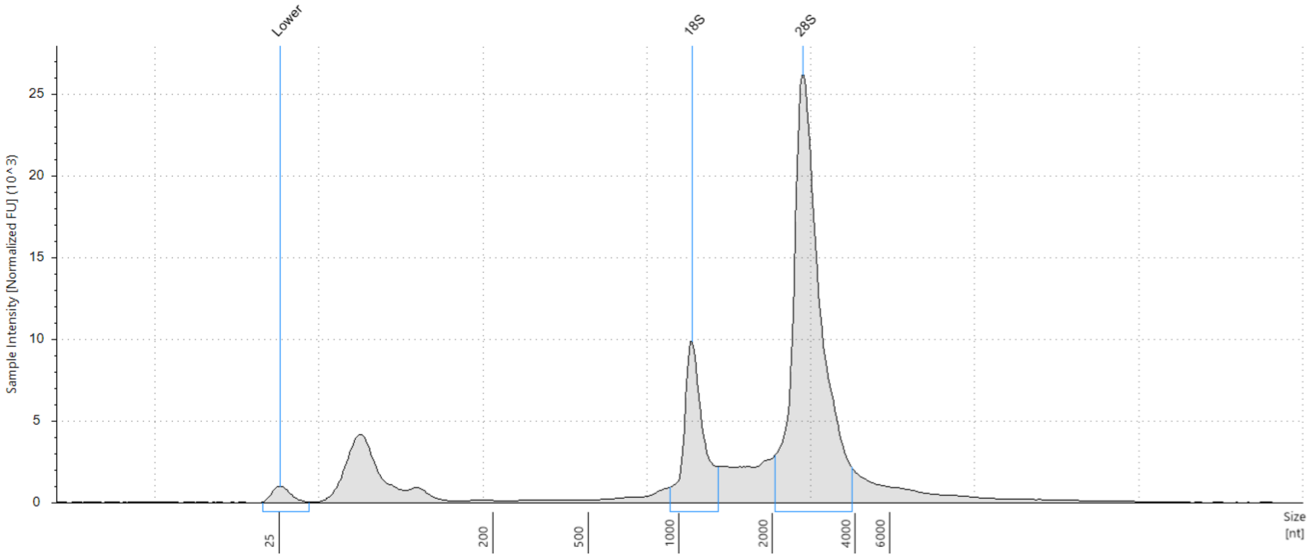

Sample Table

Well	RINe	28S/18S (Area)	Conc. [pg/µl]	Sample Description	Alert	Observations
E5	9.7	3.9	23700	WTOE B 1		

Peak Table

Size [nt]	Calibrated Conc. [pg/µl]	Assigned Conc. [pg/µl]	Peak Molarity [pmol/l]	% Integrated Area	Peak Comment	Observations
25	700	700	82400	-		Lower Marker
1090	3330	-	8970	20.50		18S
2565	12900	-	14800	79.50		28S

 © Agilent Technologies, Inc. 2017

F5: WTOE B 2

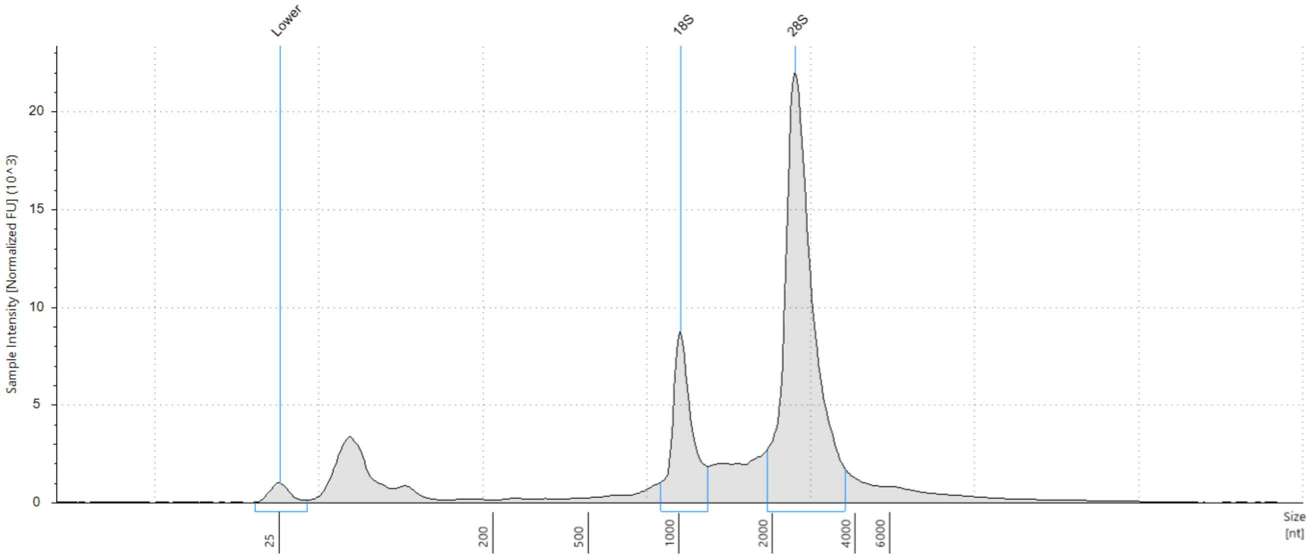

Sample Table

Well	RINe	28S/18S (Area)	Conc. [pg/µl]	Sample Description	Alert	Observations
F5	9.5	3.7	20000	WTOE B 2		

Peak Table

Size [nt]	Calibrated Conc. [pg/µl]	Assigned Conc. [pg/µl]	Peak Molarity [pmol/l]	% Integrated Area	Peak Comment	Observations
25	700	700	82400	-		Lower Marker
1006	2860	-	8370	21.13		18S
2405	10700	-	13100	78.87		28S

© Agilent Technologies, Inc. 2017

G5: WTOE PI 1

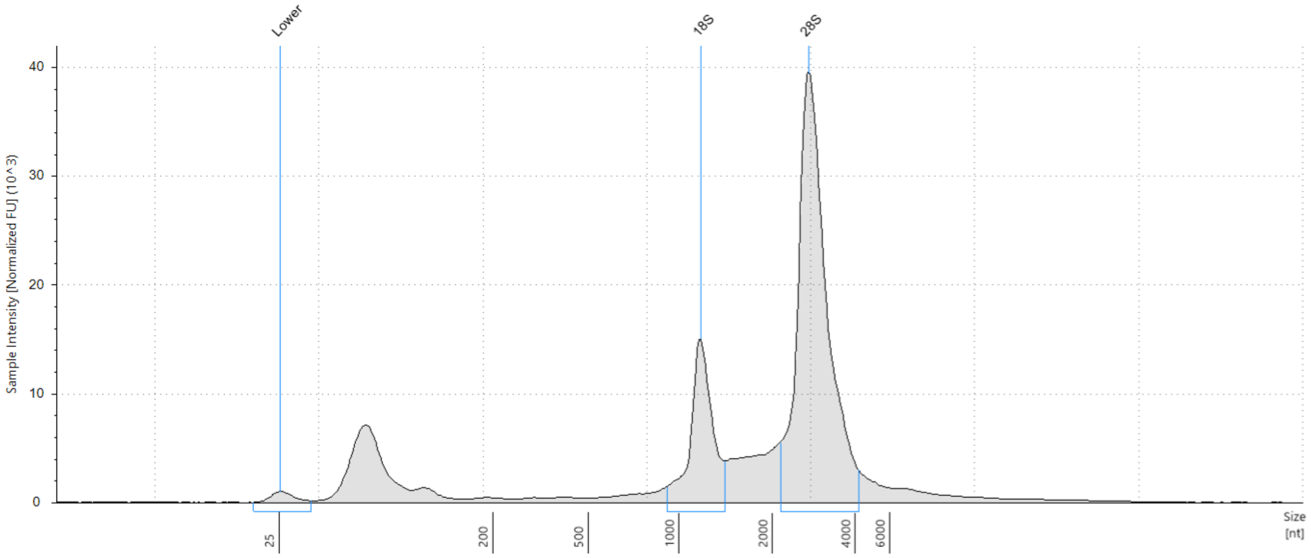

Sample Table

Well	RINe	28S/18S (Area)	Conc. [pg/µl]	Sample Description	Alert	Observations
G5	9.3	3.5	32700	WTOE PI 1	⚠	RNA concentration outside recommended range for RINe

Peak Table

Size [nt]	Calibrated Conc. [pg/µl]	Assigned Conc. [pg/µl]	Peak Molarity [pmol/l]	% Integrated Area	Peak Comment	Observations
25	700	700	82400	-		Lower Marker
1165	4870	-	12300	22.07		18S
2685	17200	-	18800	77.93		28S

H5: WTOE PI 2

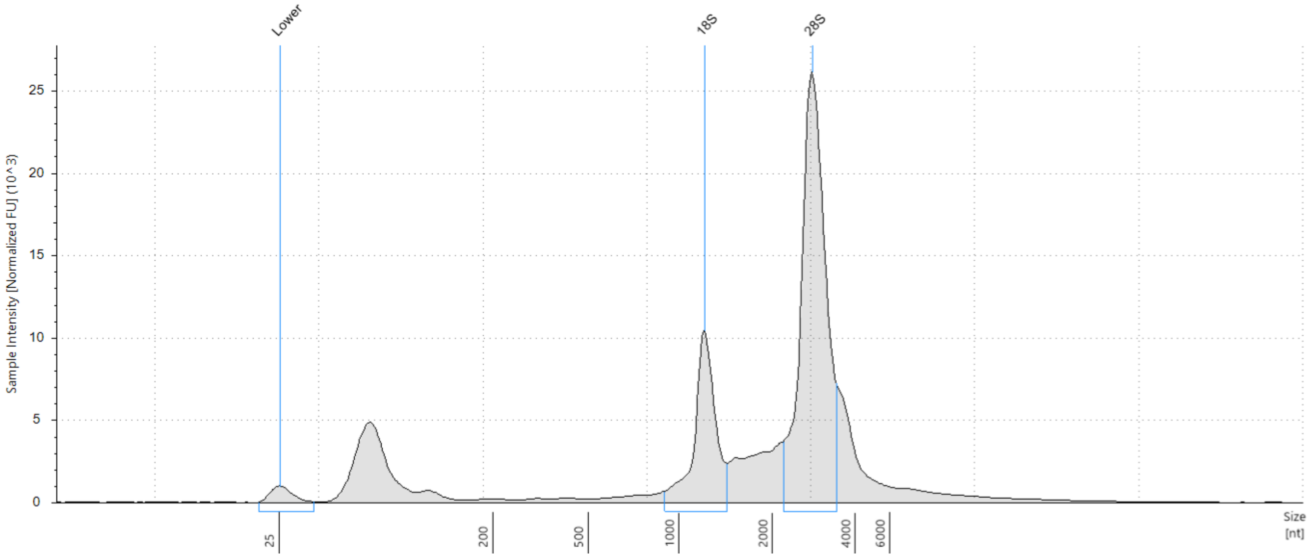

Sample Table

Well	RINe	28S/18S (Area)	Conc. [pg/µl]	Sample Description	Alert	Observations
H5	9.5	3.0	22400	WTOE PI 2		

Peak Table

Size [nt]	Calibrated Conc. [pg/µl]	Assigned Conc. [pg/µl]	Peak Molarity [pmol/l]	% Integrated Area	Peak Comment	Observations
25	700	700	82400	-		Lower Marker
1198	3410	-	8380	25.19		18S
2779	10100	-	10700	74.81		28S

A6: HYP B 1

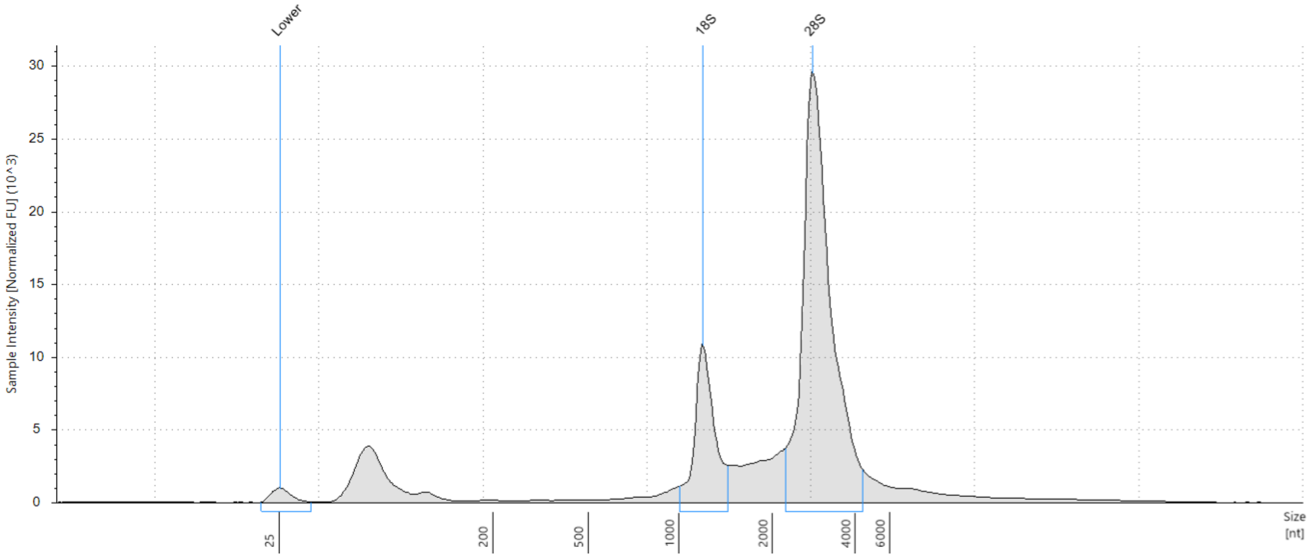

Sample Table

Well	RINe	28S/18S (Area)	Conc. [pg/µl]	Sample Description	Alert	Observations
A6	9.6	4.0	25900	HYP B 1	⚠	RNA concentration outside recommended range for RINe

Peak Table

Size [nt]	Calibrated Conc. [pg/µl]	Assigned Conc. [pg/µl]	Peak Molarity [pmol/l]	% Integrated Area	Peak Comment	Observations
25	700	700	82400	-		Lower Marker
1187	3640	-	9020	19.96		18S
2778	14600	-	15500	80.04		28S

© Agilent Technologies, Inc. 2017

B6: HYP B 2

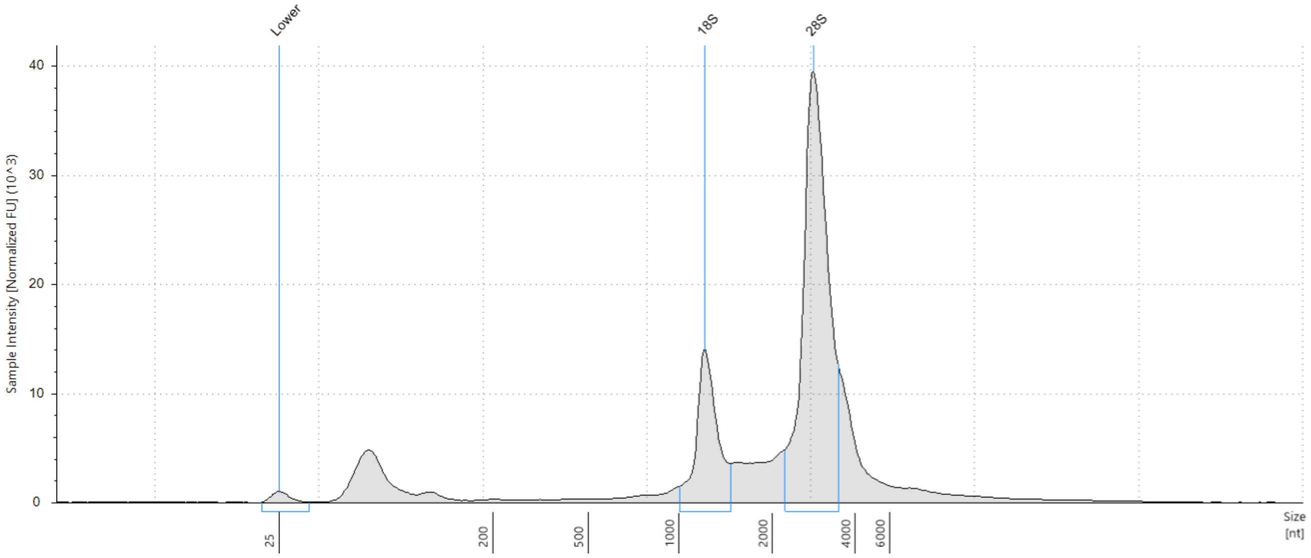

Sample Table

Well	RINe	28S/18S (Area)	Conc. [pg/µl]	Sample Description	Alert	Observations
B6	9.5	3.4	37400	HYP B 2	⚠	RNA concentration outside recommended range for RINe

Peak Table

Size [nt]	Calibrated Conc. [pg/µl]	Assigned Conc. [pg/µl]	Peak Molarity [pmol/l]	% Integrated Area	Peak Comment	Observations
25	700	700	82400	-		Lower Marker
1211	5330	-	12900	22.70		18S
2827	18100	-	18900	77.30		28S

C6: HYP PI 1

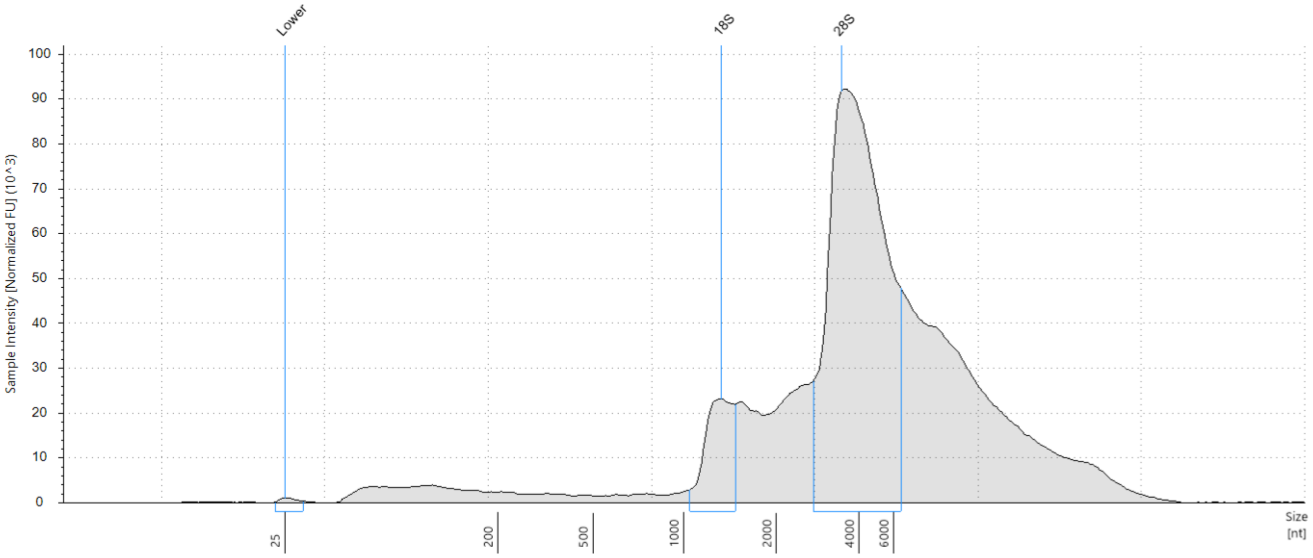

Sample Table

Well	RINe	28S/18S (Area)	Conc. [pg/µl]	Sample Description	Alert	Observations
C6	9.0	7.9	305000	HYP PI 1	⚠	RNA concentration outside recommended range for RINe; RINe edited

Peak Table

Size [nt]	Calibrated Conc. [pg/µl]	Assigned Conc. [pg/µl]	Peak Molarity [pmol/l]	% Integrated Area	Peak Comment	Observations
25	700	700	82400	-		edited Lower Marker
1323	16300	-	36200	11.19		18S edited
3446	129000	-	110000	88.81		28S edited

D6: HYP PI 2

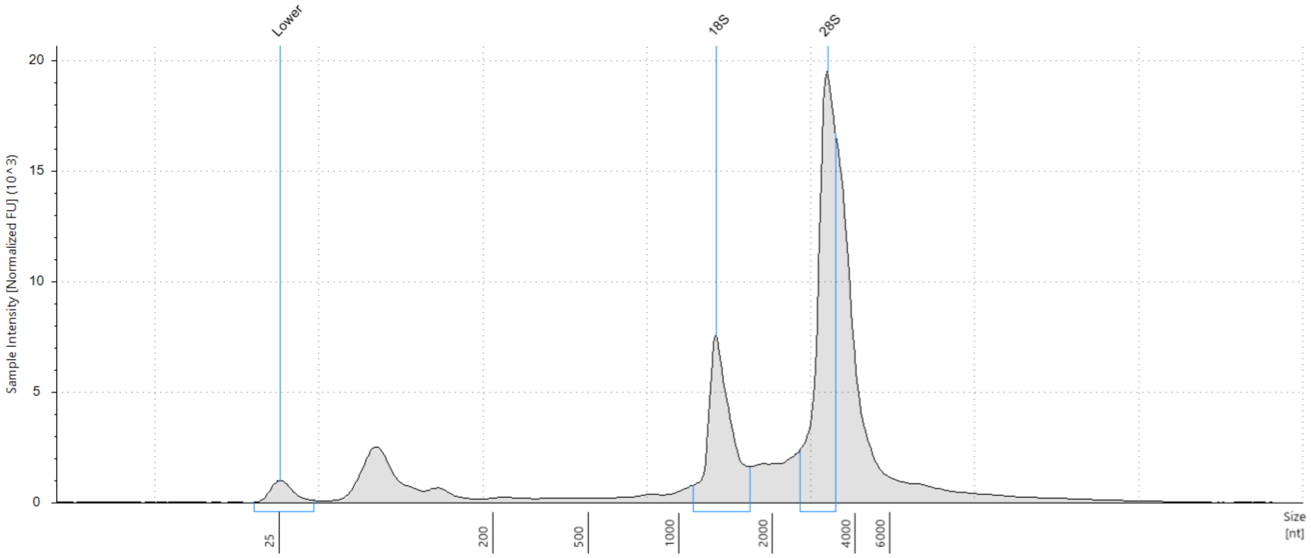

Sample Table

Well	RINe	28S/18S (Area)	Conc. [pg/µl]	Sample Description	Alert	Observations
D6	9.5	2.0	16300	HYP PI 2		

Peak Table

Size [nt]	Calibrated Conc. [pg/µl]	Assigned Conc. [pg/µl]	Peak Molarity [pmol/l]	% Integrated Area	Peak Comment	Observations
25	700	700	82400	-		Lower Marker
1303	2500	-	5640	33.43		18S
3137	4970	-	4660	66.57		28S

 © Agilent Technologies, Inc. 2017

E6: HYPOE B 1

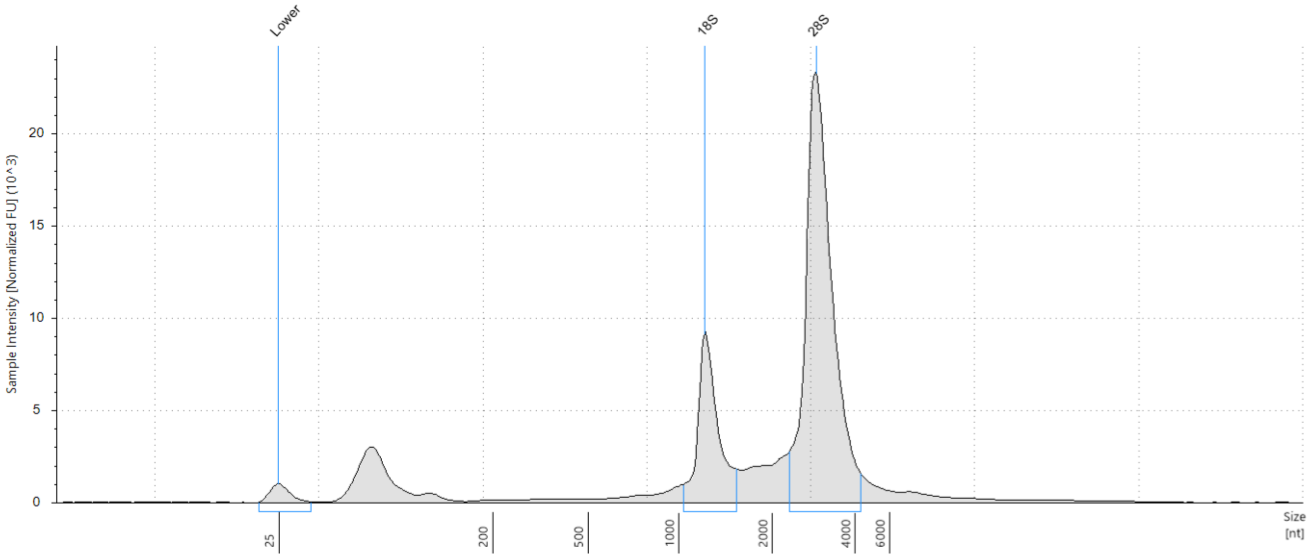

Sample Table

Well	RINe	28S/18S (Area)	Conc. [pg/µl]	Sample Description	Alert	Observations
E6	9.5	3.4	18800	HYPOE B 1		

Peak Table

Size [nt]	Calibrated Conc. [pg/µl]	Assigned Conc. [pg/µl]	Peak Molarity [pmol/l]	% Integrated Area	Peak Comment	Observations
25	700	700	82400	-		Lower Marker
1214	3090	-	7490	22.86		18S
2884	10400	-	10600	77.14		28S

F6: HYPOE B 2

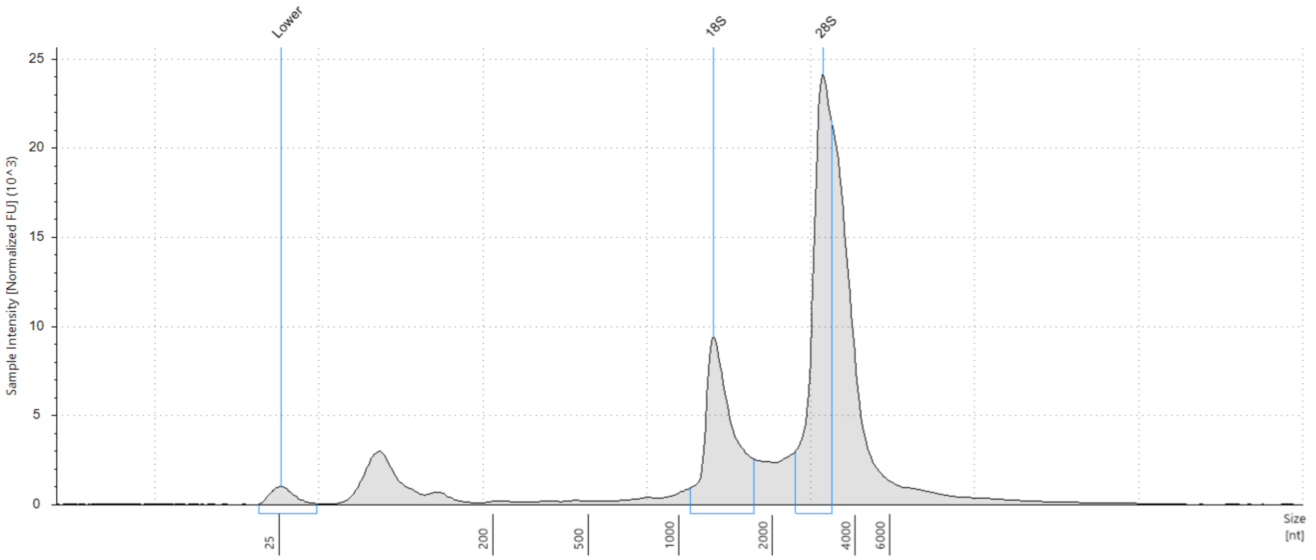

Sample Table

Well	RINe	28S/18S (Area)	Conc. [pg/µl]	Sample Description	Alert	Observations
F6	9.6	1.7	20400	HYPOE B 2		

Peak Table

Size [nt]	Calibrated Conc. [pg/µl]	Assigned Conc. [pg/µl]	Peak Molarity [pmol/l]	% Integrated Area	Peak Comment	Observations
25	700	700	82400	-		Lower Marker
1277	3580	-	8240	36.63		18S
3039	6190	-	5990	63.37		28S

© Agilent Technologies, Inc. 2017

G6: HYPOE PI 1

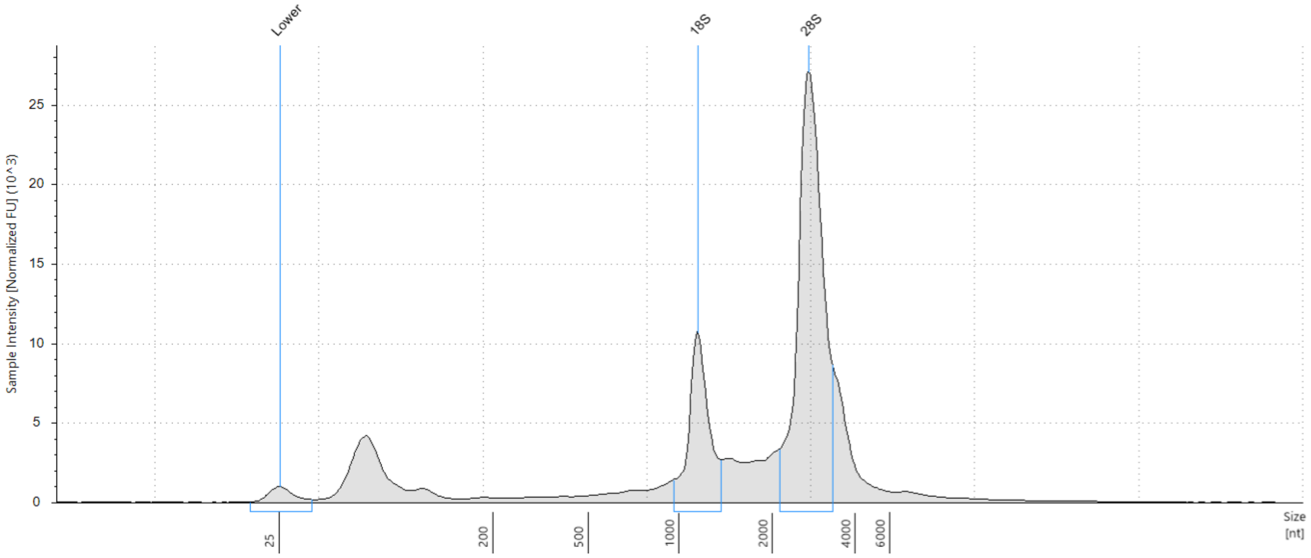

Sample Table

Well	RINe	28S/18S (Area)	Conc. [pg/µl]	Sample Description	Alert	Observations
G6	9.1	3.2	20000	HYPOE PI 1		

Peak Table

Size [nt]	Calibrated Conc. [pg/µl]	Assigned Conc. [pg/µl]	Peak Molarity [pmol/l]	% Integrated Area	Peak Comment	Observations
25	700	700	82400	-		Lower Marker
1139	2910	-	7500	23.93		18S
2687	9240	-	10100	76.07		28S

H6: HYPOE PI 2

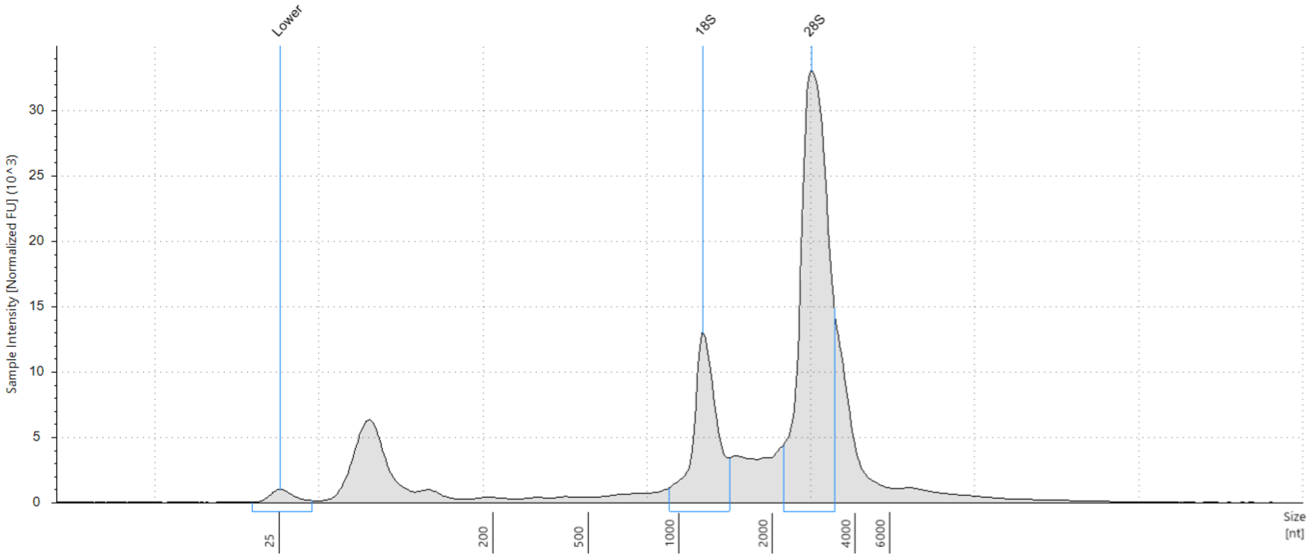

Sample Table

Well	RINe	28S/18S (Area)	Conc. [pg/µl]	Sample Description	Alert	Observations
H6	9.4	3.1	28800	HYPOE PI 2	⚠	RNA concentration outside recommended range for RINe

Peak Table

Size [nt]	Calibrated Conc. [pg/µl]	Assigned Conc. [pg/µl]	Peak Molarity [pmol/l]	% Integrated Area	Peak Comment	Observations
25	700	700	82400	-		Lower Marker
1182	4280	-	10700	24.55		18S
2753	13200	-	14100	75.45		28S

 © Agilent Technologies, Inc. 2017
