# Supplementary figures and images for: Wnt pathway inhibitors are upregulated in XLH dental pulp cells in response to odontogenic differentiation
Source: Int J Oral Sci. 2023 Feb 27;15:13. doi: 10.1038/s41368-022-00214-z (PMC9971210; doi:10.1038/s41368-022-00214-z)

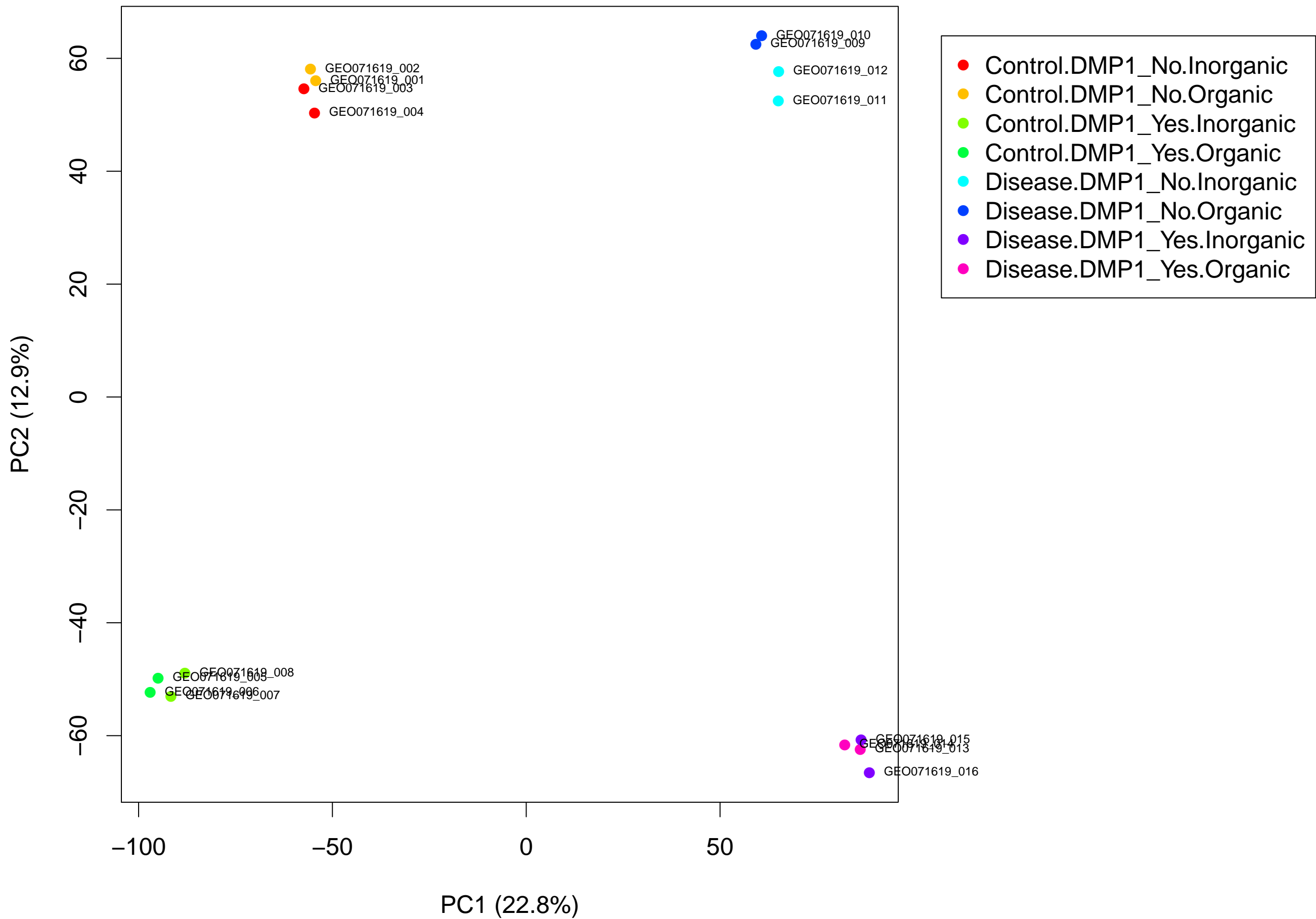

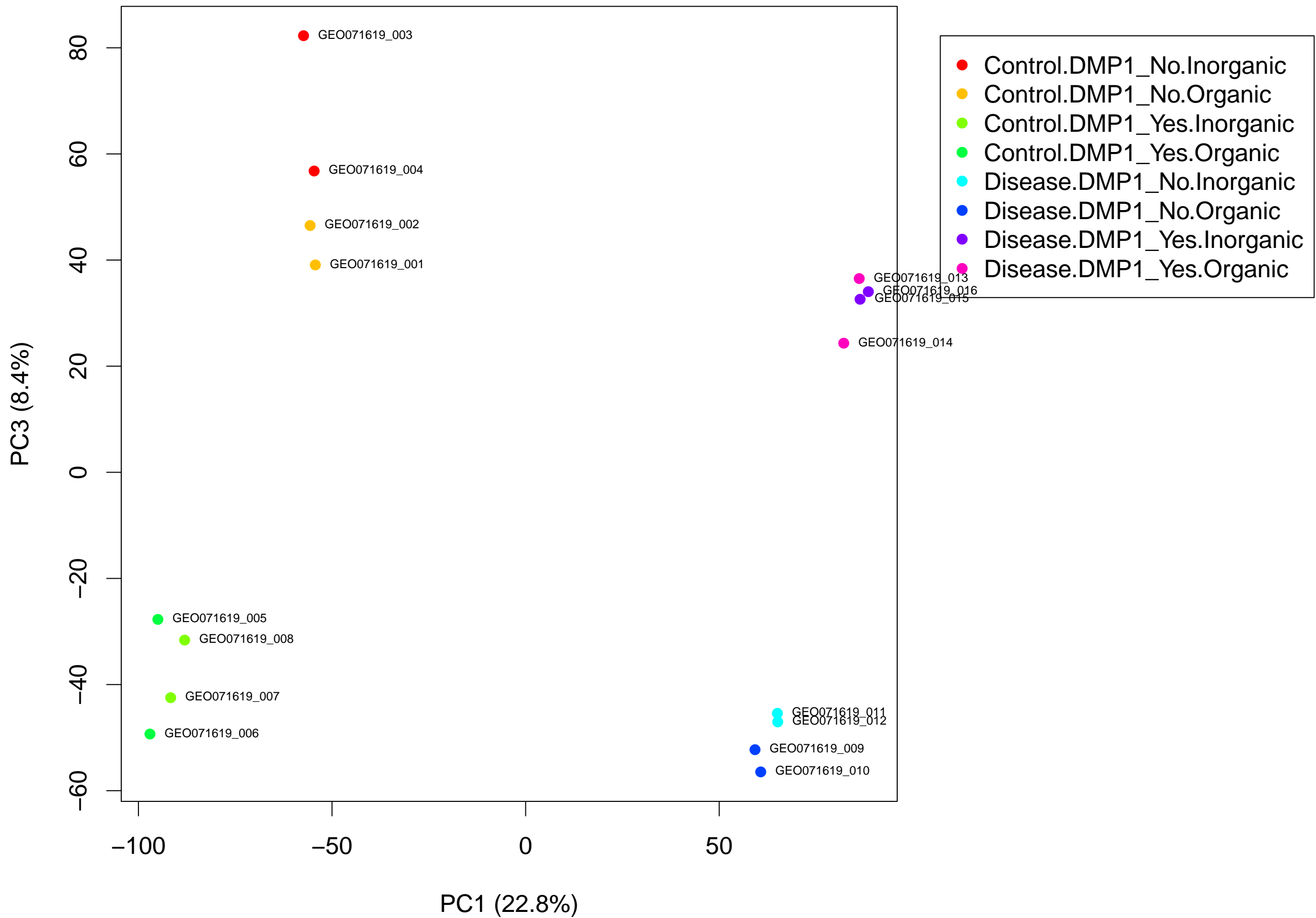

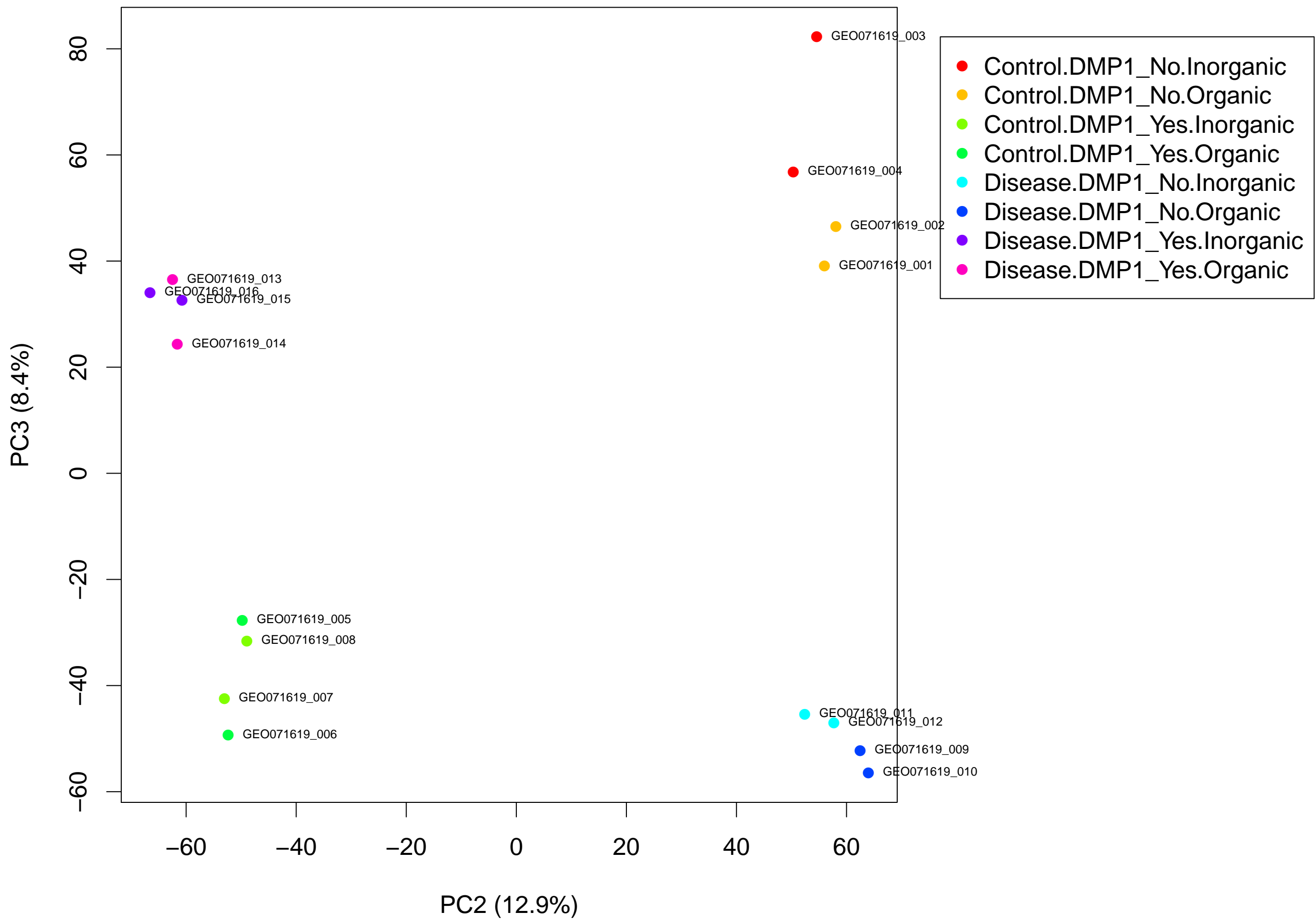

Supplement: Supplementary file 2 — Principal Component Analysis [file 41368_2022_214_MOESM2_ESM.pdf]
