# Supplementary material for: Wnt pathway inhibitors are upregulated in XLH dental pulp cells in response to odontogenic differentiation
Source: Int J Oral Sci. 2023 Feb 27;15:13. doi: 10.1038/s41368-022-00214-z (PMC9971210; doi:10.1038/s41368-022-00214-z)
Supplement: Supplementary file 3 — Supplementary Figure 1 [file 41368_2022_214_MOESM3_ESM.pptx]

## Slide 1
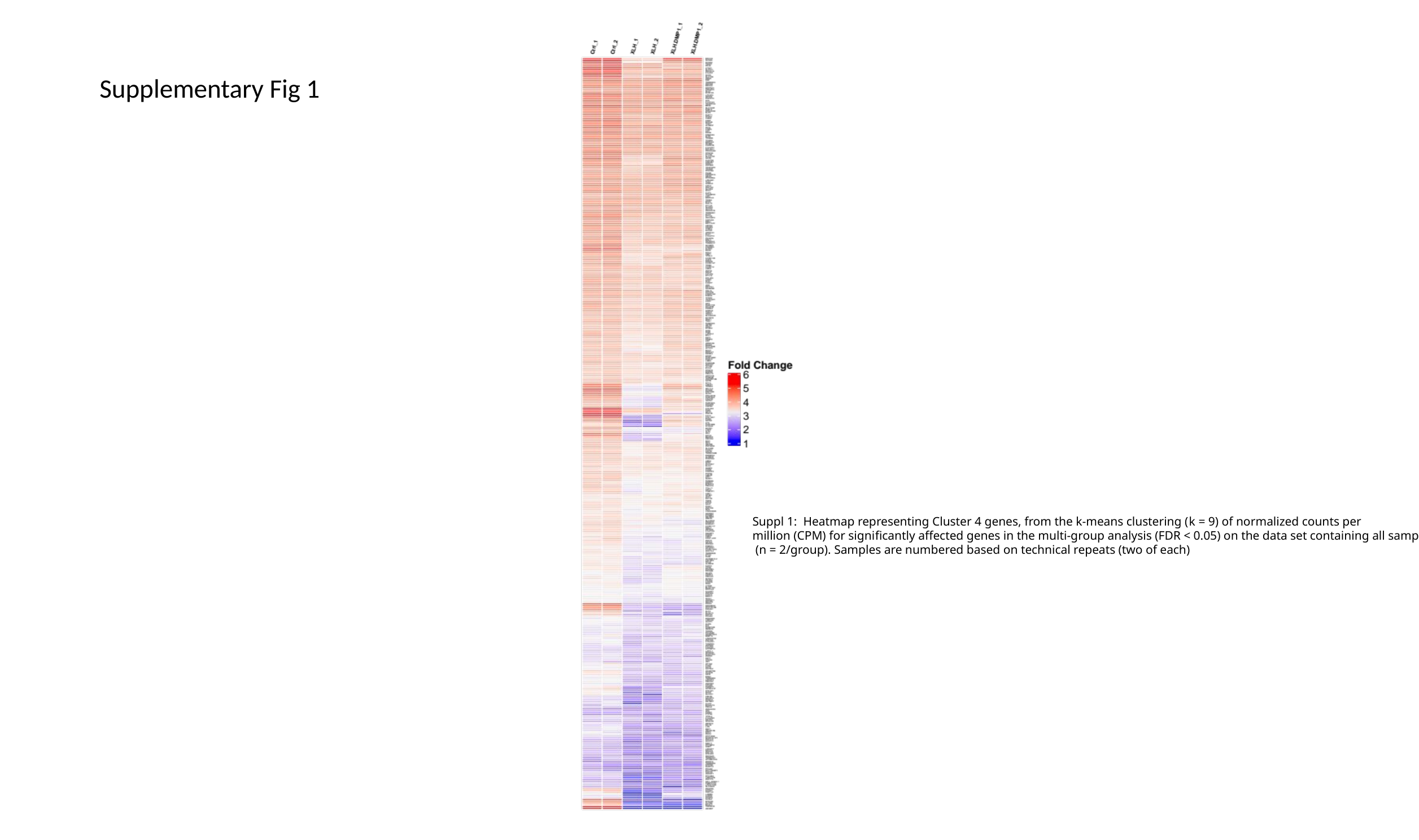

Supplementary Fig 1
Suppl 1: Heatmap representing Cluster 4 genes, from the k-means clustering (k = 9) of normalized counts per
million (CPM) for significantly affected genes in the multi-group analysis (FDR < 0.05) on the data set containing all samples
 (n = 2/group). Samples are numbered based on technical repeats (two of each)
